# Multi-sensor fusion and segmentation for autonomous vehicle multi-object tracking using deep Q networks

**DOI:** 10.1038/s41598-024-82356-0

**Published:** 2024-12-28

**Authors:** K. Vinoth, P. Sasikumar

**Affiliations:** School of Electronics Engineering, Vellore Institute of Technology, Vellore, India

**Keywords:** Multi-sensor fusion, Segmentation, Dense net (D net), Self-driving vehicles, YOLO V7 model, Energy Valley Optimizer (EVO), Engineering, Computer science

## Abstract

Autonomous vehicles, often known as self-driving cars, have emerged as a disruptive technology with the promise of safer, more efficient, and convenient transportation. The existing works provide achievable results but lack effective solutions, as accumulation on roads can obscure lane markings and traffic signs, making it difficult for the self-driving car to navigate safely. Heavy rain, snow, fog, or dust storms can severely limit the car’s sensors’ ability to detect obstacles, pedestrians, and other vehicles, which pose potential safety risks. So, we have presented a multi-sensor fusion and segmentation for multi-object tracking using DQN in self-driving cars. Our proposed scheme incorporates the handling of pipelines for camera and LiDAR data and the development of an autonomous solution for object detection by handling sensor images. An Improved Adaptive Extended Kalman Filter (IAEKF) was used for noise reduction. The Contrast enhancement was done using a Normalised Gamma Transformation based CLAHE (NGT-CLAHE), and the adaptive thresholding was implemented using an Improved Adaptive Weighted Mean Filter (IAWMF) which was used for preprocessing. The multi-segmentation based on orientation employs various segmentation techniques and degrees. The dense net-based multi-image fusion gives more efficiency and a high memory in terms of fast processing time. The Energy Valley Optimizer (EVO) approach is used to select grid map-based paths and lanes. This strategy solves complicated tasks in a simple manner, which leads to ease of flexibility, resilience, and scalability. In addition, the YOLO V7 model is used for detection and categorization. The proposed work is evaluated using metrics such as velocity, accuracy rate, success rate, success ratio, mean squared error, loss rate, and accumulated reward.

## Introduction

Multi-Object Tracking (MOT) technology enables tracking multiple moving objects within a video stream, facilitating a deeper understanding of their movements^1^. Effective MOT systems have numerous applications, including video monitoring, self-driving vehicles, and sports video analysis. Advanced MOT methods identify target objects within video frames, typically using bounding boxes, to create an automatic tracking system. A fundamental MOT system relies on two crucial components: object detection and tracking, which work together to determine target positions within each video frame and generate trajectories across frames. Rapid technological advancements are driving the increasing adoption of autonomous vehicles. To ensure safe and efficient operation, autonomous vehicles require advanced sensing technologies, perception and detection systems, and control technologies^2^. These systems utilize sensors to collect data on the surrounding environment and objects, classify and track objects, and control the vehicle accordingly. Communication technology, accurate mapping, and highway and road infrastructure optimization are essential factors. Autonomous vehicles employ various sensors, including cameras, lidar, and radar, to detect and respond to cars, pedestrians, traffic lights, signs, and other objects, ensuring driver safety and enabling seamless navigation^3^.

Autonomous vehicles rely on accurate and comprehensive environmental data to make informed driving decisions. Semantic segmentation, a deep neural network approach, identifies every pixel in camera images to provide a detailed understanding of the scene^4^. Recently, there has been growing scientific interest in autonomous driving, which, when combined with information technology, can enhance commutes, route planning, and travel times and reduce traffic congestion. Reliable self-driving systems require accurately recognizing the driving environment and vehicle conditions to ensure safety and reliability^5^. Advancements in sensor technology, exceptionally light detection and ranging (lidar), have significantly improved transportation industry monitoring due to its efficiency, speed, and adaptability^6^. Lidar technology complements radar, ultrasound, and other sensors to enhance object detection in transportation settings. Its rapid detection capabilities, independence from environmental illumination, and high resolution make it an ideal solution. Furthermore, researchers have developed an object recognition method for unmanned aerial vehicles (UAVs) called YOLOX-w, improving YOLOX-X’s performance in detecting small objects in complex backgrounds and large images^7^. This approach utilizes a shallow feature map, detection head, lightweight subspace attention module, and enhanced bounding box regression loss function.

Semantic segmentation faces challenges with fisheye images, including reduced range of view and non-rectangular shapes due to erroneous pixels. However, fisheye images simplify semantic segmentation, while object detection annotation remains cost-effective^8^. Notably, visible light cameras, commonly used in self-driving vehicles, struggle with nighttime and adverse weather conditions, compromising object detection. To address this, far-infrared images enhance nighttime and hostile environment detection. Far-infrared cameras utilize natural light from surroundings unaffected by artificial lighting to create images. Infrared radiation determines image quality, enabling early detection of pedestrians and objects^9^. Globally, traffic accidents claim 1.25 million lives and incur substantial economic costs. The World Health Organization predicts traffic accidents will become the seventh leading cause of death by 2030. Human error, driver fatigue, and limitations contribute significantly to these accidents. Autonomous vehicles, leveraging advancements in artificial intelligence, computer technology, and chip development, offer a promising solution^10–12^. Autonomous vehicles rely on LiDAR systems for environmental sensing, but these systems have limitations. LiDAR noise, vehicle noise, and background noise from various radiation sources (stars, earth, sun, atmosphere, clouds) can impair imaging^13–16^. Adverse weather conditions like heavy rain, snow, and smoke further reduce LiDAR echo detection. Researchers propose using Kalman filters and neighboring point cloud segmentation to improve LiDAR data collection in unfavorable weather. Currently, most object tracking research focuses on passenger safety features, such as airbags and pedestrian warnings, affecting Quality of Service (QoS).

Autonomous vehicles require precise environmental perception for safe navigation, particularly in challenging conditions like low visibility, severe weather, or sensor limitations. Rain, fog, or dim lighting often reduce the performance of traditional sensors like cameras and LiDAR, causing them to struggle^17^. For instance, low light conditions can cause camera-based detection to fail, and rainy or foggy conditions can compromise LiDAR data due to noise interference. To overcome these limitations, advanced multi-sensor fusion methods and robust segmentation models are essential for enhancing vehicle perception accuracy and ensuring safe operation across various scenarios. Existing approaches using deep learning models for object recognition and segmentation have shown promise but often need more sensor integration and real-time adaptability. Current methods’ reliance on single-sensor data, such as camera or LiDAR, can lead to poor object distinction and erroneous path planning, compromising autonomous navigation reliability in dynamic environments. Moreover, insufficient preprocessing can result in low-quality images and reduced segmentation accuracy, hindering object recognition and route selection. To address these challenges, multi-sensor image fusion and segmentation techniques have emerged as a solution to enhance self-driving vehicle perception systems. Combining data from multiple sensors improves environmental understanding, object detection, and navigation reliability.

### Problem statement

Autonomous vehicles rely heavily on accurate and efficient image processing and object detection to navigate safely and effectively. However, existing works in this field have several limitations that compromise the performance of these systems. This section highlights some significant problems in existing works, including a lack of image quality assessment, ineffective segmentation, inaccurate path selection, and poor Quality of Service (QoS).

#### Lack of image quality assessment

Existing works often preprocess images to enhance contrast and remove noise, but the effectiveness of segmentation still needs to be improved by missing gaps and errors in the images^31^. In the past, we have used limited pre-processing techniques like contrast enhancement and noise removal, which resulted in ineffective segmentation. To address this, a more comprehensive image quality assessment is necessary to ensure that images are high quality and suitable for segmentation.

#### Ineffective segmentation

Semantic and instance segmentation techniques have been used to segment images, but they only segment a small portion of the objects in the photos, leading to inefficient segmentation. Panoptic segmentation has also been used, but the lack of focus on the front view affects the segmentation^32^. To improve segmentation, a more effective and comprehensive approach is needed.

#### Inaccurate path selection

We have used camera images for path detection, but neglecting other images like LiDAR and FoV results in inaccurate path detection^38^. We have used segmentation techniques to detect objects at different degrees, but failing to consider the optimal degree for segmentation results in inaccurate path selection. To improve path selection, a more comprehensive approach that considers multiple images and optimal segmentation degrees is necessary.

#### Poor quality of service (QoS)

Existing works have focused on efficient map constructions and map-based path selection, but the need for more attention to destination arrival affects the QoS^44^. We have used standard camera images for segmentation, but neglecting FoV images impacts the QoS. To improve quality of service, a more comprehensive approach that considers multiple images and optimal segmentation degrees is necessary.

### Research objectives and contributions

The advancement of self-driving cars relies heavily on efficient path selection, a process that has recently faced significant challenges, particularly in image quality, segmentation, and path detection. The primary objective of this study is to achieve proper segmentation, along with accurate path and lane selection, through image pre-processing and multi-sensor image fusion in self-driving vehicles. The research aims to improve image quality by performing image quality enhancement techniques that remove noise and fill missing gaps, thereby increasing the visibility of the images. Additionally, to achieve high accuracy, the study employs multi-segmentation methods that identify narrow and smaller objects and groups of similar objects, ultimately reducing processing complexity. To enhance the Quality of Service (QoS), multi-sensor image fusion is implemented, increasing the accuracy of object detection and segmentation. Furthermore, to prevent improper lane selection, multi-object tracking is utilized to classify objects into two categories: moving and unmoving, which will enhance efficient path and lane selection. Together, these objectives contribute to improving the reliability and effectiveness of path selection in autonomous driving systems.

This study focuses on enhancing self-driving vehicle navigation through advanced image processing and multi-sensor fusion techniques. Key contributions include:

- **Image quality enhancement**: A three-stage process (noise removal, contrast enhancement, and adaptive thresholding) improves image quality, boosting segmentation accuracy.
- **Multi-segmentation**: Optimal degree segmentations (95°−30°, 30°−30°, 30°−95°) and multiple segmentations on Field of View (FoV) images enable accurate detection of narrow and small objects.
- **Multi-sensor image fusion**: Fusing camera, LiDAR, and weather images with FoV images using Dense Net (D Net) enhances segmentation accuracy and Quality of Service (QoS).
- **Accurate lane and path selection**: Grid mapping and object classification (moving/unmoving) using YOLO V7, followed by multi-object tracking, ensure reliable lane selection and reduced navigation complexity.

This research advances multi-sensor fusion by leveraging Dense Net for enhanced picture fusion, addressing existing gaps in sensor fusion and pre-processing. The proposed method provides a robust and scalable solution for dependable autonomous navigation.

### Paper organizations

This paper is structured as follows: Sect. 2 provides a comprehensive review of the existing literature, clearly identifying gaps in the current state-of-the-art and setting the stage for our contributions. Section 3 presents our proposed methodology. This section includes diagrammatical representations, mathematical formulations, and pseudo-code examples to provide a clear and detailed explanation of our approach. Section 4 presents our experimental results. This section is divided into subsections detailing the dataset used, the simulation setup, a comparative analysis of our approach against existing methods, and a discussion of our findings. Finally, Sect. 5 concludes the paper, summarizing our key contributions and outlining directions for future research.

### Related works

Recent advancements in autonomous vehicle technology have focused on enhancing motion planning, control, and path prediction to ensure safety and efficiency in dynamic environments. Various techniques, including hierarchical controllers, potential field models, and deep learning algorithms, have been proposed to improve obstacle avoidance, lane recognition, and overall decision-making. This related work section reviews key contributions in these areas, highlighting innovative strategies designed to optimize the performance of autonomous systems.

Advanced autonomous driving systems heavily rely on effective object detection methods, and numerous studies have sought to address this challenge. For instance, the Comprehensive Autonomous Driving Recognition and Learning Dataset (CARL-D) has been employed for self-driving car object identification^18^. Similarly, the KITTI benchmark suite has facilitated object identification from video sequences captured in over 100 cities. In these applications, four cameras mounted on autonomous vehicles preprocess and segment images, with ResNet utilized for end-to-end panoptic annotation segmentation. The resulting segmented images are then subjected to bounding box annotation for object detection, employing various deep learning techniques, including Faster Region-based Convolutional Neural Network (R-CNN), Region Proposal Network (RPN), Feature Pyramid Network (FPN), ResNet, Visual Geometry Group (VGG), You Only Look Once (YOLOv4), and Darknet53. Despite implementing deep neural networks for destination detection, many studies still need to adequately address efficient map construction for accurate destination arrival, leading to potential decision-making failures and Quality of Service (QoS) issues. Modern improvements in zero-shot object detection highlight the challenges in recognizing novel objects within computer vision^19^. Traditional methods often fall short, necessitating innovative strategies that combine machine learning with human expertise. One such strategy is the Human-in-the-Loop (HITL) approach, which integrates deep learning models, including convolutional neural networks (CNNs), with iterative human input from annotators. This methodology enables adaptive model refinement based on human annotations, enhancing the recognition and localization of unseen objects in visual data.

To enhance tracking capabilities, a method employing information fusion was proposed^20^. This approach leverages the Visual Object Classes (VOC 2012) dataset to analyze monocular camera images captured before the vehicle for target detection. The process involves generating and validating a target hypothesis through faster R-CNN classification to identify candidate regions. Furthermore, techniques like Online Hard Example Mining (OHEM) have been incorporated to optimize the candidate region’s loss function. Although these improvements in detection can reduce radar false negatives and positives, the reliance on raw images as input introduces additional complexity. It can compromise accuracy due to issues like noise and gaps in data. In^21^, the authors propose an enhanced semantic perception system for autonomous driving, incorporating four area view cameras, one narrow Field of View (FoV) camera, and five Light Detection and Ranging (LiDAR) scanners. The collected images undergo preprocessing, including motion correction for 3D objects, before being segmented into various degrees using CNN for applications such as route object recognition and heavy rain conditions. To improve city segmentation^22^, suggests incorporating semantic visuals into the cityscape dataset. This dataset features enhanced contrast and semantic segmentation using VGG19, enabling the identification of urban elements such as buildings, poles, roads, and vehicles. While this technique improves semantic segmentation, deep learning methods can lead to slower object detection due to the extensive training time required. A memory-augmented neural network approach for dynamic segmentation of self-driving vehicle imagery is presented in^23^. Utilizing datasets like New York University Depth V2 (NYUDv2), PASCAL Visual Object Classes Context, and challenging traffic data, this method employs Long Short-Term Memory (LSTM) networks alongside Faster R-CNN, for instance, segmentation. The memory-augmented network effectively combines original and segmented images, enabling it to process more data efficiently and improve the accuracy of target area detection. The proposed work lacks focus on object tracking, which can lead to inaccurate lane detection. The^24^addresses maritime traffic situation awareness by leveraging ship imaging trajectories derived from various maritime data sources, including automatic identification systems and surveillance videos. To enhance the quality of marine images, the authors apply a dark channel prior model to eliminate fog, resulting in clear, high-resolution ship images. They employ a scale adaptive kernel correlation filter for ship tracking, generating raw imaging trajectories refined by removing anomalies through curve-fitting and downsampling techniques. The proposed framework is evaluated using three distinct maritime scenarios, demonstrating its effectiveness in accurately extracting ship imaging trajectories for improved traffic situation awareness. Maritime traffic safety is enhanced in^25^ by developing a video-based ship trajectory extraction system. It implements a three-step framework: initially, it determines ship positions using a combination of YOLOX and CenterNet models; next, it examines ship contours utilizing a deep snake module; and finally, it tracks successive ship trajectories with an upgraded Bytetrack algorithm. This method enhances situational awareness for ship crews, enabling more effective maneuvering in maritime settings.

In^26^, a fused residual network with feature pyramids is introduced for pedestrian recognition in self-driving cars. This approach incorporates a residual network dropout layer to enhance model generalizability and feature selection and alignment modules that refine feature extraction. A cascaded autofocus query module accelerates pedestrian detection while preprocessing improves image contrast. However, the effectiveness of segmentation is often compromised by image noise. To address improvements in object detection, the paper presents M-Net, a deep learning model distinguished by its spatial resolution and broad receptive field^27^. The model utilizes pyramid mid-pooling modules for multiscale context aggregation and employs multipath feature extraction to enhance segmentation accuracy. Experiments demonstrate high-accuracy image segmentation using VGG19 for both segmentation and object detection. However, this method requires significant training time, which may slow object detection due to less desirable layers. In^28^, a dynamic algorithm-based multi-template object tracking correlation method is proposed. The Adaptive Dynamic Multi-Template Association Filter (ADMTC) addresses four tracking challenges by utilizing local binary pattern features in Hue, Saturation, and Value (HSV) color space, which helps minimize image noise from light variations. The ADMTC maintains tracking precision with adaptable templates and proves stable and robust across varied environments. This model employs vehicle kinematics and Euclidean solenoids to establish a driving risk field, gathering data on traffic participants, lane lines, and signals to refine distance calculations. Applying the principle of least action enhances intersection passage efficiency and safety, thereby increasing the success rate over time.

The authors of^29^suggest a method for proposing a LiDAR outlier filter that effectively removes snow particles from raw LiDAR point clouds using Adaptive Density Outlier Removal (AGDOR) filters, as demonstrated on challenging winter driving datasets. An intensity threshold and outlier elimination technique enhances the filter’s effectiveness, improving accuracy for autonomous driving systems under harsh conditions. A novel multi-sensor fusion scheme is introduced in^30^, addressing security concerns related to non-differentiable target cameras and LiDAR sensing systems. This research features a Multi-Scale Fusion (MSF) algorithm to enhance perception in autonomous vehicles. The findings indicate that the approach successfully protects various object types. While objects are detected using deep neural networks, a lack of differentiation among detected objects can lead to improper path selection. Additionally, this study employs a Neuro-fuzzy aggregation deep learning framework for data fusion, integrating information from multiple sources using fuzzy measurements. A neural model for steering control and obstacle detection is also presented, illustrating a robust operating system and prototype for autonomous vehicle control. The approach involves preprocessing images through noise removal, contrast enhancement, and adaptive thresholding, all contributing to improved segmentation accuracy^31^. In^32^, the authors examine object detection challenges in severe fog by testing the visibility of four thermal targets at fixed positions. Key factors influencing image quality include target intensity, background contrast, sensor-to-target temperature distances, camera angle, fog density, and object sensor distance. The research emphasizes issues with semantic segmentation, which could have counted objects within the images accurately. A monocular camera-based anchor-free 3D object detection system called Key Point is introduced to address these challenges. This system utilizes geometric constraints to project object localization from the world coordinate system onto the 2D image plane as 3D geometric center points. Self-adapting elliptical Gaussian filters enhance the system’s ability to manage varying object shapes. At the same time, a deformable convolutional network improves resilience against affine transformations, yielding favorable results in practical driving scenarios^33^. The research also presents a unique Self-Driving Car (SDC-Net) framework that utilizes multitask neural networks to integrate different input representations from a CARLA simulator. This system encompasses crash prevention, route selection, and emergency braking technology, facilitating safe navigation through various driving conditions. It leverages a benchmark dataset with diverse vehicle scenarios, supporting efficient pathways and automated emergency braking^34^. A real-time grid map occupancy detection study is also detailed in^35^. This method uses adjusted occupancy grid maps as input and output for bounding boxes surrounding vehicles, implemented via YOLOv2 and YOLOv3 for both image and LiDAR point cloud data. The proposed system enhances vehicle detection by integrating current and previous occupancy grid maps. It optimizes route planning, although the fusion of FoV and LiDAR images needs to be improved, potentially leading to erroneous path selection.

In autonomous driving, researchers are increasingly focusing on utilizing LiDAR scans to enhance the capabilities of self-driving neural networks by providing route information for small country roads^36^. By integrating LiDAR with camera data, efforts aim to improve the robustness and adaptability of driving systems, particularly in adverse weather conditions. The research is based on a substantial real-world dataset collected from extensive rural driving, emphasizing the need to address prediction error sizes to establish a comprehensive performance metric. Additionally, the authors propose an autonomous vehicle sensor fusion method utilizing surveillance cameras to enhance traffic monitoring. The Enhanced Autonomous and Intelligent Driving (EAID) framework implements a fused sensor-based collision detection system (FSCDS) along with deep learning techniques to improve AV navigation, validated through accurate car tests in various driving scenarios^37^. Moreover, researchers have developed a YOLOX-based network model, which concentrates on multiscale item detection in complex scenes^38^. This model integrates an object contextual feature fusion module that enhances multiscale perception and employs a scaling factor to reduce object loss while improving overall recognition. The architecture utilizes a multi-head self-attention block to assess query similarities, resulting in precise bounding boxes for roadside objects, with the softmax function ensuring relevant network outputs^39^. Subsequently, the work employs convolutional neural networks (CNNs) for segmentation, which can lead to sluggish convergence and high complexity due to numerous layers and data requirements. The Center Fusion approach facilitates 3D object recognition using radar and video data, employing a frustum-based technique to align radar detections with image features^40^. However, the lower resolution of radar sensors than cameras complicates matching processes, potentially resulting in false positives and negatives. The “Camera Radar Fusion-Net” (CRF-Net) architecture integrates data from vehicle cameras and radar sensors^41^. This innovative approach addresses radar processing challenges with the “BlackIn” training method, although it does not clarify how CRF-Net accounts for issues related to noisy radar data. Moreover, the significance of a large dataset for self-driving scene identification is emphasized, featuring various real-world traffic scenarios. The authors propose combining labels with a deep data exchange network framework to tackle the dataset’s imbalance and enhance classification performance on misclassified samples. The availability and potential expansion of the Driving Scene dataset are pivotal for improving multi-class classification methods in autonomous driving^42^, though the effectiveness of this approach in resolving data imbalance remains unclear. This research introduces a novel Multi-Sensor Fusion Object Detection framework^43^, an innovative convolutional neural network (CNN) model designed for efficient obstacle detection in autonomous driving. By combining visual and LiDAR data, this framework achieves exceptional accuracy and real-time performance on resource-constrained edge devices. Its modular architecture and refinement techniques enable robust detection of vehicles and pedestrians in diverse scenarios.

In^44^, the authors present a hybrid sensor fusion approach for self-driving vehicles, integrating vision, LiDAR, and radar data to create comprehensive environmental maps for optimal decision-making and actuator control. Utilizing the FCNx deep learning framework and Extended Kalman Filter (EKF) on an edge computing device, this method enables effective and cost-efficient sensor fusion. However, the study does not quantify the accuracy or performance of the fusion methodology. Additionally, redesigning lane lines and distance metrics enhances deep neural network training, with the TuSimple and CULane datasets demonstrating reliable lane line recognition. The proposed network features fewer parameters than previous models, making it suitable for embedded hardware like the PX^45^. It effectively detects lane lines in challenging conditions such as occlusions, corners, shadows, and traffic congestion but does not address detection in adverse weather or complex road layouts. In another study^46^, camera images and LIDAR point clouds are fused to develop a Fully Convolutional Network (FCN) for road detection. This system employs dynamic integration of LIDAR and camera processing through trainable cross-links across all layers. The proposed cross-fusion FCN outperforms single-modality networks and existing fusion techniques on the KITTI road benchmark, demonstrating its robustness in visually demanding scenarios and highlighting the advantages of sensor integration for road detection.

This work^47^proposes a post-impact motion planning and stability control technique for autonomous cars. It suggests an enabling motion planning technique for post-impact scenarios by merging the artificial potential field and polynomial curve with obstacle avoidance. We subsequently create a hierarchical controller, comprising an upper and a lower controller, to track the intended motion. The higher controller uses a time-varying linear quadratic regulator to determine the intended generalized forces. The lower controller properly coordinates the actuators using a torque allocation approach based on nonlinear optimization to achieve the intended generalized forces. The author in^48^ proposed a three-dimensional potential field (TriPField) model. Specifically, they solve the specified Laplace equation using boundary conditions in ellipsoidal coordinates, modeling the surrounding vehicles as ellipsoids. They design the AV’s velocity profiles to enhance processing efficiency and minimize the path search space, following the development of a nonparametric GVF that captures multi-vehicle interactions. Finally, they create a local path planning framework using the TriPField, incorporating model predictive control to account for vehicle kinematic restrictions.

To learn lane recognition and path prediction (PP) in autonomous driving from start to finish, the author in^49^suggests a lightweight UNet that makes use of depthwise separable convolutions (DSUNet). Additionally, they combine a PP algorithm with a convolutional neural network (CNN) to create a simulation model (CNN-PP). This allows for the dynamic, qualitative, and quantitative evaluation of CNN’s performance in a host agent car driving alongside other agents in real-time autonomous mode. Compared to UNet, DSUNet has a 1.61 quicker inference speed and a 5.12 lighter model. This research^50^suggests using deep learning (DL) based algorithms in the control layer of an autonomous car. This research explicitly compares the outcomes of Deep Reinforcement Learning (DRL) algorithms such as Deep Deterministic Policy Gradient (DDPG) and Deep Q-Network (DQN). Using a DRL method, this study aims to create a trained model that can provide control orders to the vehicle so that it can travel correctly and efficiently along a predetermined path. This article^51^ describes the construction of a hierarchical dynamic drifting controller (HDDC) that tracks the overall route evenly within and beyond stability limitations while performing both drifting and standard cornering actions. In particular, the HDDC consists of the actuator regulating layer, vehicle motion control layer, and route tracking layer. We have validated the algorithmically flexible first layer using MPC and LQR. Its purpose is to create the appropriate states and provide precise route tracking. We suggest using the dynamic drifting inverse model to combine drifting and conventional cornering control in the second layer. The third layer can execute the steering system and wheel speed control to meet the relevant directives.

### Proposed method

**Fig. 1:**
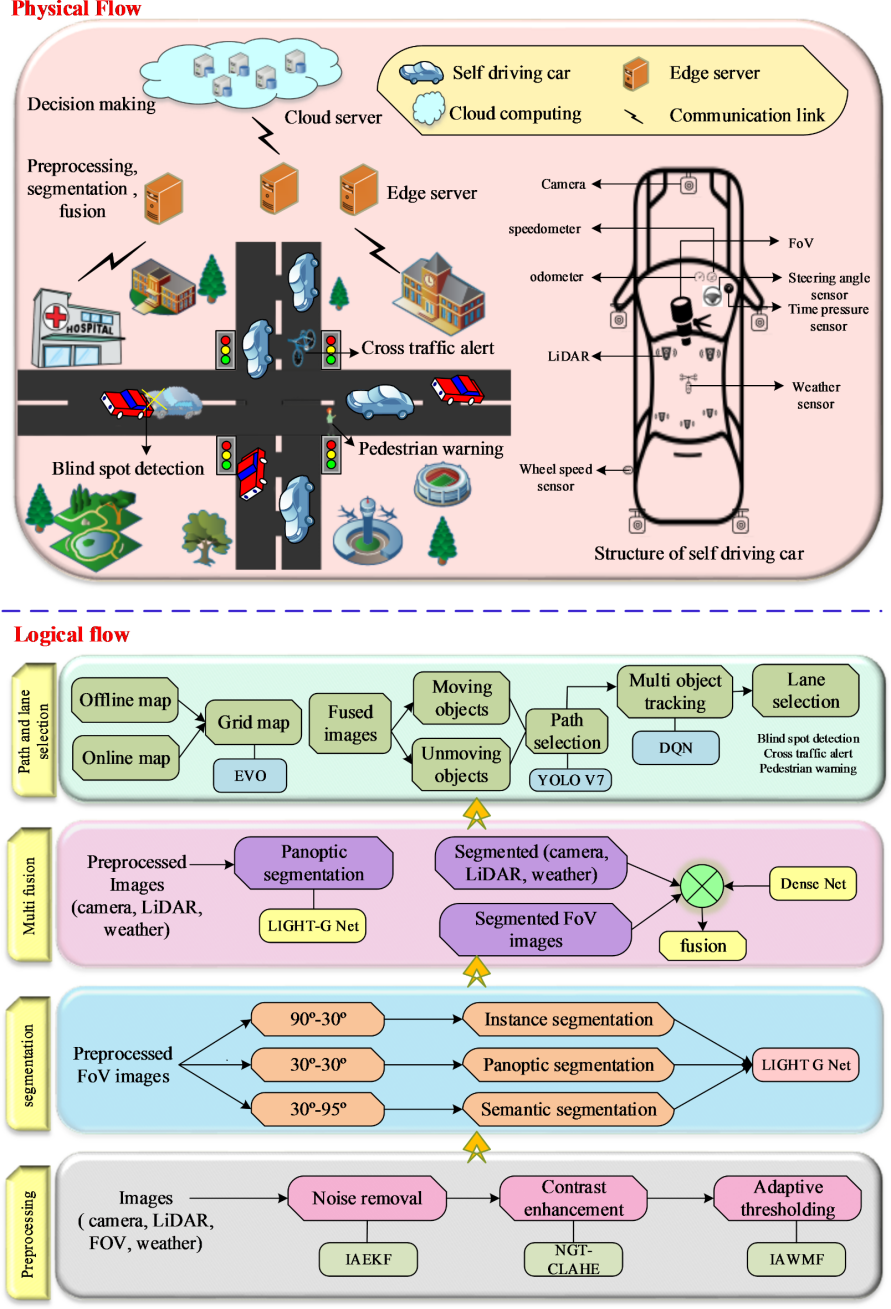
Overall Design of the proposed DQN Model.

In our research, we mainly focus on accurate path and lane selection in self-driving cars by performing pre-processing and optimal path selection. The KITTI dataset which consists of camera images, LiDAR images, weather images, and FoV images are considered. The major entities involved in the proposed work are sensors, edge servers, and the cloud. Cloud computing and edge servers are employed to reduce storage burden and adaptable computation. Further, image processing and segmentation are performed in the edge server which offers high-speed data processing and reduces energy consumption. The architecture of the proposed Deep Q-Network (DQN) model, designed to facilitate multi-object tracking and segmentation for autonomous vehicles in challenging environments, is illustrated in Fig. 1. of this study. This architecture encompasses several key steps, beginning with image quality enhancement, which refines raw sensor data through techniques such as adaptive thresholding, contrast enhancement using Normalized Gamma Transformation (CLAHE), and noise reduction with the Improved Adaptive Extended Kalman Filter (IAEKF). These enhancements are crucial for ensuring the provision of high-quality inputs for subsequent processing. A significant aspect of this stage is the Improved Adaptive Weighted Mean Filter (IAWMF), which effectively reduces noise without compromising image characteristics. By adjusting the filtering intensity based on the characteristics of each pixel, IAWMF assigns greater weights to pixels likely to contain critical information, resulting in cleaner and more accurate images than traditional filtering methods.

Following this enhancement, the multi-segmentation phase divides images into various fields of view (FoV) and orientations, allowing for precise identification of smaller or more confined objects in the environment. This segmentation is achieved using the Light G Net, a network specifically designed for high-speed processing. Light G Net facilitates real-time segmentation in autonomous vehicles by employing a lightweight convolutional framework with dense connections, which ensures efficient feature retention while minimizing computational overhead. To further enhance the segmented images from this step, multi-sensor fusion is implemented using a dense net-based architecture. This process integrates data from multiple sensors, including cameras and LiDAR, to create a comprehensive fused representation of the vehicle’s surroundings. Finally, the Energy Valley Optimizer (EVO) and YOLO V7 models are utilized for dynamic and accurate real-time path planning, enabling the detection and tracking of objects for precise lane and path selection.

The proposed work consists of four major processes namely,

- Image quality enhancement.
- Orientation based multi segmentation.
- Dense Net-based multi-image fusion.
- Grid map-based path and lane selection.

### Image quality enhancement

Image quality enhancement is mainly implemented to improve the quality of the images by performing several processes such as noise removal, contrast enhancement, and adaptive thresholding which are described as follows. The preprocessing of the KITTI dataset involved a multi-step approach to enhance image quality and ensure robust multi-sensor fusion. Initially, we employed the Improved Adaptive Extended Kalman Filter (IAEKF) to minimize ambient noise and improve sensor reliability. Subsequently, adaptive thresholding and normalized gamma transformation-based CLAHE (NGT-CLAHE) were applied to fill gaps and boost contrast, enhancing visibility. To simulate severe weather conditions, such as low light levels, fog, and rain, we modified the sensor data to mimic scenarios where visibility and object identification are compromised. This included reducing intensity and contrast to mimic low light conditions, adding noise and blur to simulate fog and rain, and altering color and texture to replicate adverse weather effects. By testing our model in these simulated adverse conditions, we ensured its reliability for safe navigation in real-world scenarios.

#### IAEKF-based noise removal

In this part, the enhanced EKF is used to predict noise removal. For this, an augmented Kalman state vector with the variation coefficient is represented as Y=$\:[{p\_1\dots\:p\_r\:\:a]}^{T}$. The model error may contain p. Thus, the state equation becomes Eq. (1)

1 $$\:\dot{Y}\left(t\right)=\text{m}\left(Y\left(t\right)\right)=\left({V}^{-1}\right)\left(b\left(t\right)-N{\dot{p}}_{0}^{\dot{p\left(t\right)}}\left(t\right)-a\left(t\right){S}_{p}\left(t\right)\right)+K\left(t\right)$$

m(.) is a nonlinear function, and K(t) represents process noise with zero mean and covariance matrix P(t). To simplify, wind loading is added to the process noise. (3) Eq. 

2 $$\:\dot{Y\left(t\right)}=m\left(Y\left(t\right)\right)=\left({V}^{-1}\left(-N\dot{p}{\left(t\right)}_{0}^{\dot{p\left(t\right)}}-a\left(t\right){S}_{p}\left(t\right)\right)\right)+K\left(t\right)=I\left(Y\left(t\right)\right)+K\left(t\right)$$

The nonlinear function is denoted by I(.). Only a few accelerometers can measure the car’s position. Thus, the measurement equation contains q DoFs of measurement information.

3 $$\:{X}_{k+1}=\left[\begin{array}{ccccc}{0}_{1\times\:r}&\:{\phi\:}_{1\left({b}_{a}^{\left(1\right)}\right)}&\:\vdots&\:{\phi\:}_{r}\left({b}_{a}^{\left(1\right)}\right)&\:0\\\:\vdots&\:\vdots&\:\ddots\:&\:\vdots&\:\vdots\\\:{0}_{1\times\:r}&\:{\phi\:}_{1}\left({b}_{a}^{\left(q\right)}\right)&\:\dots\:&\:{\phi\:}_{r}\left({b}_{a}^{\left(q\right)}\right)&\:0\end{array}\right]I\left({Y}_{s+1}\right)+{C}_{s+1}={\phi\:}_{s+1}I\left({Y}_{s+1}\right)+{C}_{s+1}$$

where $\:{\text{X}}_{(\text{k}+1)}$is the observation vector containing the observed acceleration response at discrete time instant t=(s+1)$\:\varDelta\:$t, $\:\varDelta\:$t is the sampling time step, and$\:{\:b}_{a}^{\left(i\right)}$ is the i-th accelerometer location. The mode shape function of mode r is $\:{\phi\:}_{r}\left(\right)$, and the measurement noise has a zero mean and a covariance matrix $\:{A}_{(\text{s}+1)}$=$\:{F\left[{C}_{(s+1)}{-C}_{(s+1)}\right]}^{T}.$ Eq. (2)

4 $$\:{X}_{s+1}=W\left({Y}_{s+1}\right)+{C}_{s+1}$$

The first-order Taylor series expansion can linearize Eqs. (2) and (4) and$\:{\stackrel{\sim}{Y}}_{\left(s+1|s\right)}$ respectively.

5 $$\:I\left(Y\right)\approx\:I\left({\widehat{Y}}_{s|s}\right)+{Y}_{s|s}{H}_{s|s}\left(Y-{\widehat{Y}}_{s|s}\right),{H}_{s|s}=\frac{\partial\:I\left(Y\right)}{\partial\:Y}{|}_{Y={\widehat{Y}}_{s|s}}$$

6 $$\:W\left({Y}_{s+1}\right)\approx\:W\left({\stackrel{\sim}{Y}}_{s+1|s}\right)+{G}_{s+1|s}\left({Y}_{s+1}-{\stackrel{\sim}{Y}}_{s+1|s}\right),{G}_{s+1|s}=\frac{\partial\:W\left(Y\right)}{\partial\:Y}{|}_{Y={\stackrel{\sim}{Y}}_{s+1|s}}$$

Where $\:{\widehat{Y}}_{s|s}$ is the predicted state vector at time instant $\:t=s\varDelta\:t$, including the calculated noise reduction variation coefficient, and $\:{\stackrel{\sim}{Y}}_{(s+1|s)}$is the projected state vector at t=(s+1)$\:\varDelta\:t$ in Eq. (5) (6). The $\:{H}_{\left(s\right|s)}$ and $\:{G}_{(s+1|s)}$matrices are calculated as given Eq. (7) (8) below.

7 $$\:{H}_{s|s}=\left[-\begin{array}{ccc}{0}_{r\times\:r}&\:{U}_{r\times\:r}&\:0\\\:{\widehat{a}}_{s|s}{V}^{-1}W&\:{-V}^{-1}N&\:-{V}^{-1}W{\widehat{p}}_{s|s}\\\:0&\:0&\:0\end{array}\right]$$

8 $$\:{G}_{s+1|s}={\phi\:}_{s+1}\left[-\begin{array}{ccc}{0}_{r\times\:r}&\:{U}_{r\times\:r}&\:0\\\:{\stackrel{\sim}{a}}_{s+1|s}{V}^{-1}W&\:{-V}^{-1}N&\:-{V}^{-1}W{\stackrel{\sim}{p}}_{s+1|s}\\\:0&\:0&\:0\end{array}\right]$$

Like the original EKF, the IAEKF predicts and corrects the augmented Kalman state vector. The time update process predicts the state vector $\:{Y}_{(s+1|s)}$ in the prediction phase in Eq. (9).

9 $$\:\:{\stackrel{\sim}{Y}}_{s+1|s}={\widehat{Y}}_{s|s}+{\int\:}_{s\varDelta\:t}^{(s+1)\varDelta\:t}I\left({\widehat{Y}}_{t|s}\right)dt$$

The prediction error is $\:\:{\stackrel{\sim}{c}}_{(s+1|s)}$=$\:{\text{Y}}_{(\text{s}+1)}$-$\:{\stackrel{\sim}{Y}}_{s+1|s}$, and its covariance matrix is$\:{\stackrel{\sim}{Q}}_{s+1|s}$=F[$\:{\stackrel{\sim}{c}}_{s+1|s}{\stackrel{\sim}{c}}_{s+1|s}^{T}$]. Additionally, $\:{\stackrel{\sim}{Q}}_{s+1|s}$is expressed as

10 $$\:{\stackrel{\sim}{Q}}_{s+1|s}={\lambda\:}_{s+1}{\varphi\:}_{s|s}{\widehat{Q}}_{s|s}{{\Phi\:}}_{s|s}^{T}{\lambda\:}_{s+1}^{T}+{P}_{s+1}$$

Here $\:{\varphi\:}_{s|s}$is the state transition matrix, and $\:{\widehat{\:Q}}_{s|s}$is the covariance matrix of the prediction error at the previous time step in Eq. (10). The inclusion of the fading-factor matrix,$\:\:{\lambda\:}_{s+1}$odulates the influence of past state information, thereby slowing the decay of useful information. This adjustment ensures that the filter remains sensitive to recent changes in the system, which improves the accuracy of the state estimation.

Where$\:{{\Phi\:}}_{s|s\approx\:{U}_{2r+1}}+\varDelta\:t{H}_{s|s}$,$\:{\widehat{\:Q}}_{s|s}={\widehat{c}}_{s|s}{\widehat{c}}_{s|s}^{T}$,$\:{\:U}_{2r+1}$ is a 2r+1 identity matrix, and $\:{\lambda\:}_{s+1}$is the fading-factor matrix at t=(s+1)$\:{\Delta\:}$t. Adding $\:{\lambda\:}_{s+1}$ slows information decay and tracks parameter changes. In the improvement stage, the measurement brings up-to-date process and replaces the prediction$\:{\stackrel{\sim}{Y}}_{s+1|s}$with the measured value in (11):

11 $$\:{\stackrel{\sim}{Y}}_{s+1|s+1}={\stackrel{\sim}{Y}}_{s+1|s}+{W}_{s+1}[{X}_{s+1}-W\left({\stackrel{\sim}{Y}}_{s+1|s}\right)]$$

In this update equation $\:{\stackrel{\sim}{Y}}_{s+1|s+1}$is the corrected state vector after incorporating the new measurement$\:{X}_{s+1}$The Kalman gain matrix $\:{W}_{s+1}$determines how much the new measurement influences the state update. This gain is computed using the predicted error covariance $\:{\widehat{Q}}_{s+1|s}$ which has already been adjusted for the state variation via the fading-factor matrix in Eq. (10).

$\:{\stackrel{\sim}{Y}}_{s+1|s+1}$is the valued state vector at t=(s + 1)$\:{\Delta\:}$t, and$\:{W}_{s+1}$ is the Kalman increase matrix which is generated as Eqs. (12) (13),

12 $$\:{W}_{s+1}={\stackrel{\sim}{Q}}_{s+1|s}{G}_{s+1|s}^{T}{\left({G}_{s+1|s}{\stackrel{\sim}{Q}}_{s+1|s}{G}_{s+1|s}^{T}+{A}_{s+1}\right)}^{-1}$$

The state estimation error covariance matrix is very easy to compute.

13 $$\:{\widehat{Q}}_{s+1|s+1}=\left({U}_{2r+1}-{W}_{s+1}{G}_{s+1|s}\right){\widehat{Q}}_{s+1|s}$$

**Fig. 2:**
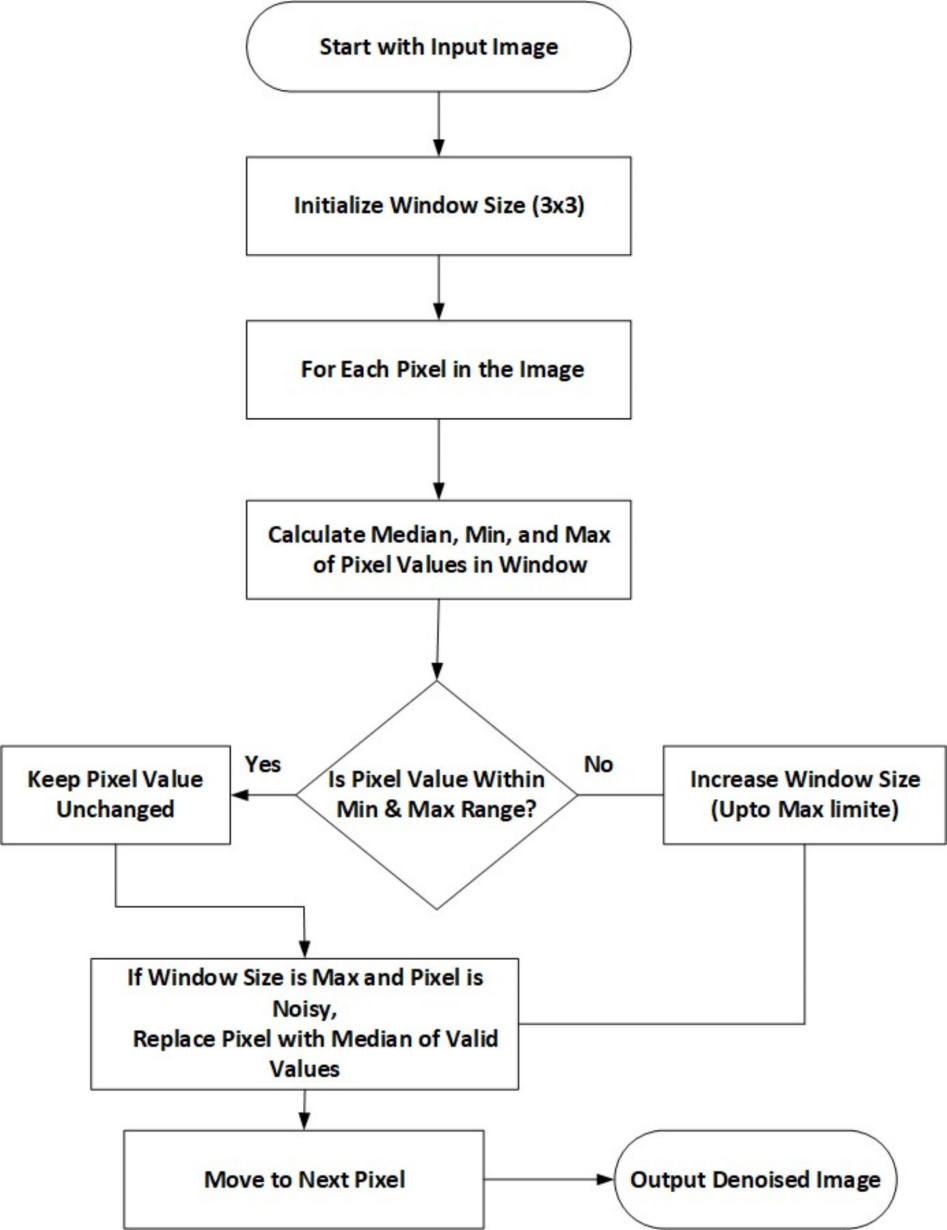
Flowchart for Improved Extended Kalman Filter (IAEKF).

The IAEKF method comprises two main parts. The error covariance matrices in Eq. (2) would be reorganized step-by-step through IAEKF execution since they include uncertain wind loading. Secondly, Eq. (10) demonstrates that the fading-factor matrix choice determines the state estimation accuracy. The estimated physical parameter vector should improve when $\:{\lambda\:}_{s+1}$is updated over time. Thus, the fading-factor matrix is updated. Figure 2. represents the flowchart for IAEKF.

The Improved Extended Kalman Filter (IAEKF) is used in self-driving vehicles’ perception systems to anticipate and eliminate noise. With this method, model errors and outside disturbances are taken into account by using an enhanced Kalman state vector with a variation coefficient. A nonlinear function that is represented by the state equation and linearized by a first-order Taylor series expansion accounts for wind loading and process noise. When the state vector is updated based on fresh observations and noise is corrected using the Kalman gain matrix, the prediction phase approximates the state vector. To adjust for changes in parameters over time, the error covariance matrix is progressively improved by adding a fading-factor matrix. This technique guarantees improved detection and tracking performance under a range of circumstances by increasing the precision of noise reduction and the dependability of the sensor data fusion process.

#### Update of the error covariance matrices

Online error covariance matrices are inferred using the Bayesian probabilistic method in this portion. An error parameter matrix is defined as $\:{\theta\:}_{s+1}$=$\:{\left[{P}_{s+1}{A}_{s+1}\right]}^{T}$. According to Bayes’ theorem, reducing an objective function O($\:{\theta\:}_{s+1}$) yields the best approximation of the error parameter matrix.

14 $$\:{\widehat{\theta\:}}_{s+1|s+1}=arg\underset{{\theta\:}_{s+1}}{\text{min}}O\left({\theta\:}_{s+1}\right)$$

Where$\:O{(\theta\:}_{s+1})$, as defined by Eq. (15)

15 $$\:O{(\theta\:}_{s+1})={e}_{0}+\frac{1}{2}[In\left|{\stackrel{\sim}{Q}}_{s+1|s}^{X}\right|+\frac{1}{{u}_{0}}{\left({\theta\:}_{s+1}-{\widehat{\theta\:}}_{s|s}\right)}^{T}{\left({\widehat{Q}}_{s|s}^{\theta\:}\right)}^{-1}\left({\theta\:}_{s+1}-{\widehat{\theta\:}}_{s|s}\right)+{\epsilon\:}_{s+1}^{T}{\left({\stackrel{\sim}{Q}}_{s+1|s}^{X}\right)}^{-1}{\epsilon\:}_{s+1}$$

Where $\:{\widehat{Q}}_{s+1}^{\theta\:}$ is the latter covariance matrix of the noise parameters, $\:{\epsilon\:}_{s+1}$ is the output remaining arrangement which is expressed as $\:{\epsilon\:}_{s+1}$ =$\:{X}_{s+1}-{\stackrel{\sim}{X}}_{s+1|s}$. $\:{\stackrel{\sim}{X}}_{s+1|s}$can be calculated using $\:{\stackrel{\sim}{X}}_{s+1|s}=s\left({\stackrel{\sim}{Y}}_{s+1|s}\right)$ and$\:{\stackrel{\sim}{Q}}_{s+1|s}^{Y}$ is the measurement prediction covariance which is calculated using Eq. (16)

16 $$\:{\stackrel{\sim}{Q}}_{s+1|s}^{X}={G}_{s+1|s}{{\Phi\:}}_{s|s}{\widehat{Q}}_{s|s}{{\Phi\:}}_{s|s}^{T}{G}_{s+1|s}^{T}+{G}_{s+1|s}{\widehat{Q}}_{s|s}{G}_{s+1|s}^{T}+{\widehat{A}}_{s|s}$$

#### Update of the fading-factor matrix

Equation (10) indicates that the fading-factor matrix considerably affects the time-varying physical parameter estimation accuracy. The fading-factor matrix is selected adaptively in this Eqs. (17), (18), (19):

17 $$\:{\lambda\:}_{s+1}=diag[{1}_{1\times\:2r},{\mu\:}_{s+1}]$$

Where

18 $$\:{\mu\:}_{s+1}=\left\{\begin{array}{c}{{\mu\:}_{0,s+1},\mu\:}_{0,s+1},\ge\:0\\\:1,{\mu\:}_{0,s+1}<0\end{array}\right.$$

And

19 $$\:{\mu\:}_{0,s+1}=\sqrt{\frac{tr\left[{Z}_{s+1}\right]}{{tr[V}_{s+1}]}}$$

The Trace operator is represented as tr[.]. The $\:{Z}_{s+1}$ is calculated as Eq. (19)

20 $$\:{Z}_{s+1}={C}_{s+1}-{G}_{s+1|s}{\widehat{P}}_{s+1|s+1}{G}_{s+1|s}^{T}+s{\widehat{A}}_{s+1|s+1}$$

Where $\:s\ge\:1$ is the selected weakening factor in Eq. (20) (21), and

21 $$\:{C}_{s+1}=\left\{\begin{array}{c}{\epsilon\:}_{1}{\epsilon\:}_{1}^{T},s=0\\\:\frac{\beta\:{C}_{s}+{\epsilon\:}_{s+1}{\epsilon\:}_{s+1}^{T}}{1+\beta\:},s\ge\:1\end{array}\right.$$

The forgetting factor is β(0 < β) and $\:{V}_{s+1}$can be approximated by the Eq. (22)

22 $$\:{V}_{s+1}=[{\stackrel{\sim}{Q}}_{s+1|s}^{ti}-{\widehat{P}}_{s+1|s+1}]{G}_{s+1|s}^{T}{G}_{s+1|s}$$

State prediction covariance without fading-factor matrix (23) is $\:{\stackrel{\sim}{Q}}_{s+1|s}^{ti}$.

23 $$\:{\stackrel{\sim}{Q}}_{s+1|s}^{ti}={{\Phi\:}}_{s|s}{\widehat{Q}}_{s|s}{{\Phi\:}}_{s|s}^{T}+{\widehat{P}}_{s+1|s+1}$$

#### Contrast enhancement

Based on extensive experimental data, we chose CLAHE to enhance the visual contrast. CLAHE uses the contrast-limiting technique aimed at all neighborhoods to generate an alteration function. However, if the damaged image is too bright, the corrected image may have an uneven contrast. To fix this, we added the gamma function to CLAHE. The gamma modification helps improve contrast and brightness in the CLAHE images. The most popular image color correction method is the ‘grey world’. This method presupposes that the RGB average value color images in all channels have equal quality, i.e., a normal color image is grey.$\:{\:R}_{av}$, $\:{G}_{av}$, and $\:{B}_{av}$ indicate the average RGB color channel values of a picture, while$\:{\:R}_{c}$,$\:\:{G}_{c}$, and $\:{B}_{c}$ represent the corrected RGB color channel values.

The grey-level gamma function translation function relates the pixel luminance to a numerical value. Thus, it brightens photos. The original image q’s pixel values are in the range of [0,1], Let$\:{q}_{\text{c}\text{o}\text{r}\text{r}\text{e}\text{c}}$be the gamma-corrected image, a be a positive constant parameter, and d be the brightness parameter. Thus,

24 $$gamma =\:{q}_{\text{c}\text{o}\text{r}\text{r}\text{e}\text{c}}=d{q}^{\gamma\:}$$

Equation (24) shows that the transformation function can be modified. The projected image will be darker if the function is raised. Thus, the dynamic range should cover its natural interval. In this work, the NGC reduces the gamma correction’s downsides. The NGC equation is given as (25) :

25 $$\:{L}_{NGC}=\frac{[{q}_{\text{c}\text{o}\text{r}\text{r}\text{e}\text{c}}-\text{m}\text{i}\text{n}({q}_{\text{c}\text{o}\text{r}\text{r}\text{e}\text{c}}t\left)\right]}{[\text{max}\left({q}_{\text{c}\text{o}\text{r}\text{r}\text{e}\text{c}}\right)-\text{m}\text{i}\text{n}({q}_{\text{c}\text{o}\text{r}\text{r}\text{e}\text{c}}\left)\right]}$$

$\:{L}_{NGC}$ is the normalized gamma function output indices. The NGC’s complete dynamic range normalization reduces brightness and improves contrast.

- Color correction by adjustment of the a and b components.

26 $$\:\left\{\begin{array}{c}{R}_{c}=R\frac{{G}_{av}}{{R}_{av}}\\\:{G}_{c}=G\\\:{B}_{c}=B\frac{{G}_{av}}{{B}_{av}}\end{array}\right.$$

The transition of picture channels in RGB color space is not linear. Hence, pixels of the same hue but different lightness would have different colors after revision with (26), resulting in distorted RGB color correction.

Lab color space uses linear color fluctuation in the a and b components to convert colors. Thus, it may solve the issues better. $\:{A}_{av}$and $\:{B}_{av}$ are the average a and b component values in the Lab color space, while $\:{A}_{c}$and $\:{B}_{c}$are the corrected values (27). The lab color space adjusts the image color to

27 $$\:\left\{\begin{array}{c}{A}_{c}=a-{A}_{av}\\\:{B}_{c}=b-{B}_{av}\end{array}\right.$$

- Description of our method.

NGCCLAHE contains seven separate operation phases, with steps 2–7 established on CLAHE:

##### Step 1

Adapt raw pictures from RGB to lab color space using these formulas (depicts images as an m-by-n-by-3 numeric array, with the elements indicating the red, green, and blue color channels’ intensities in Eqs. (28–31).

28 $$\:\left\{\begin{array}{c}S=116f\left(\frac{V}{100}\right)-16\\\:a=500\left[f\left(\frac{U}{95.047}\right)-f\frac{V}{100}\right]\\\:b=200\left[f\left(\frac{V}{100}\right)-f\left(\frac{W}{108.883}\right)\right]\end{array}\right.$$

Where

29 $$\:\left[\begin{array}{c}U\\\:V\\\:w\end{array}\right]=\left[\begin{array}{ccc}0.4124&\:0.3576&\:0.1805\\\:0.2126&\:0.7152&\:0.0722\\\:0.0193&\:0.1192&\:0.9505\end{array}\right]\left[\begin{array}{c}{R}^{\text{*}}\\\:{G}^{\text{*}}\\\:{B}^{\text{*}}\end{array}\right]$$

30 $$\:f\left(t\right)=\left\{\begin{array}{c}{t}^{\raisebox{1ex}{$1$}\!\left/\:\!\raisebox{-1ex}{$3$}\right.}\:ift>{\left(\frac{6}{29}\right)}^{3}\\\:\frac{1}{3}{\left(\frac{29}{6}\right)}^{2}t+\frac{4}{9}\end{array}\right.$$

31 $$\:\left\{\begin{array}{c}{R}^{\text{*}}=gamma\left(\frac{R}{255}\right)\\\:{G}^{\text{*}}=gamma\left(\frac{G}{255}\right)\\\:{B}^{\text{*}}=gamma\left(\frac{B}{255}\right)\end{array}\right.$$

R, G, and B are R, G, and B channel pixel value indexes. S, a, and b are channel pixel value indices.

##### Step 2

Correct the contrast of the L module of the pictures utilizing the NGC function (25).

32 $$\:{S}_{NGC}=\frac{[{S}_{\text{c}\text{o}\text{r}\text{r}\text{e}\text{c}}-\text{m}\text{i}\text{n}({S}_{\text{c}\text{o}\text{r}\text{r}\text{e}\text{c}}\left)\right]}{[\text{max}\left({S}_{\text{c}\text{o}\text{r}\text{r}\text{e}\text{c}}\right)-\text{m}\text{i}\text{n}({S}_{\text{c}\text{o}\text{r}\text{r}\text{e}\text{c}}\left)\right]}\:\:\:\:\:\:\:\:\:\:\:\:\:\:\:\:\:\:\:\:\:\:\:\:\:\:\:\:\:\:\:\:\:\:\:\:\:\:\:\:\:\:\:\:\:\:\:\:\:$$

Where

$$\:{S}_{\text{c}\text{o}\text{r}\text{r}\text{e}\text{c}}=d{S}^{\gamma\:}$$

In our experiment, we assumed that d = 1 and = 0.5.

##### Step 3

Tile contrast-adjusted S component picture (32). This study uses K = M = 8.

##### Step 4

Calculate the tile histogram using a CDF. The relevant CDF is given by.

33 $$\:{f}_{j,i}\left(n\right)=\frac{M-1}{K}\sum\:_{k=0}^{n}{p}_{j,i}\left(k\right)\:\:\:\:\:\:\:\:\:\:\:\:\:\:\:\:\:\:\:\:\:\:\:\:\:\:\:\:\:\:\:\:\:\:\:\:\:\:\:\:\:\:\:\:\:\:\:\:\:\:\:\:\:\:\:\:\:\:\:\:\:\:\:\:\:\:\:\:\:\:\:\:\:\:\:\:\:\:$$

Where $\:{p}_{j,\:i}$(k) are histogram indexes for pixel k; K, M, and *n* = 0, 1, 2,…, M −1 are tile pixel counts (33); and j and$\:{i}_{ar}$ are pixel k location indexes.

##### Step 5

Using the following equation, calculate the value of the clip limit.

34 $$\:\beta\:=\frac{K}{M}\left(1+\frac{\alpha\:}{100}\left({G}_{max}-1\right)\right)\:\:\:\:\:\:\:\:\:\:\:\:\:\:\:\:\:\:\:\:\:\:\:\:\:\:\:\:\:\:\:\:\:\:\:\:\:\:\:\:\:\:\:\:\:\:\:\:\:\:\:\:\:\:\:\:$$

α is the clip factor and $\:{G}_{max}$ is the maximum allowable slope (34).

##### Step 6

Keep histograms below their clip limit β and cut those over it.

##### Step 7

Distribute the updated histogram values to appropriate bins.

##### Step 8

Use these mapping functions to calculate the new pixel values from the updated histogram distribution and contrast-limited region locations.

35 $$\:{q}_{new}^{inner}=\frac{{g}_{vb}}{{g}_{vt}+{g}_{vb}}\left(\frac{{g}_{hr}}{{g}_{hl}+{g}_{hr}}{f}_{j-1,i-1}{q}_{old}^{inner}+\frac{{g}_{hl}}{{g}_{hl}+{g}_{hr}}{f}_{j,i-1}{q}_{old}^{inner}\right)+\frac{{g}_{vt}}{{g}_{vt}+{g}_{vb}}\left(\frac{{g}_{hr}}{{g}_{hl}+{g}_{hr}}{f}_{j-1,i}{q}_{old}^{inner}+\frac{{g}_{hl}}{{g}_{hl}+{g}_{hr}}{f}_{j,i}{q}_{old}^{inner}\right)\:\:\:\:\:\:\:\:\:\:\:\:\:\:\:\:\:\:\:\:\:\:\:\:\:\:\:\:\:\:\:\:\:\:\:\:\:\:\:\:\:\:\:\:\:\:\:\:\:\:\:\:\:\:\:\:\:\:\:\:\:\:\:\:\:\:\:\:\:\:\:\:\:\:\:$$

36 $$\:{q}_{new}^{boder}=\frac{{g}_{vb}}{{g}_{vt}+{g}_{vb}}{f}_{j,i-1}{q}_{old}^{boder}+\frac{{g}_{vt}}{{g}_{vt}+{g}_{vb}}{f}_{j,i}{q}_{old}^{boder}\:\:\:\:\:$$

37 $$\:{q}_{new}^{corner}={f}_{j,i}{q}_{old}^{corner}\:\:\:\:\:\:\:\:\:\:\:\:\:\:\:\:\:\:\:\:\:\:\:\:\:\:\:\:\:\:\:\:\:\:\:\:\:\:\:\:\:\:\:\:\:\:\:\:\:\:\:\:\:\:\:\:\:\:\:\:\:\:\:\:\:\:\:\:\:\:\:$$

where $\:{q}_{new}$and $\:{q}_{old}$ are indices of the new pixel value at location (u, v) in an area of (j, i), (j, i-1), (j-1, i), and (j-1, i-1) after and before the redistribution of its associated histogram, respectively (35–37). The region indexes are inner, border, and corner. In vertical and horizontal directions, the indexes,$\:\:{g}_{vb}$,$\:{g}_{vt}$, $\:{g}_{hr}$and$\:\:{g}_{hl}$ of the preceding equations are the distances of the pixel at location (u, v) to pixels (j, i), (j, i-1), and (j-1, i-1).

##### Step 9

Fix the a and b chromatic components with and (27).

##### Step 10

Convert the modified Lab images to RGB using these Eqs. 38 and 39.

38 $$\:\left[\begin{array}{c}R\\\:G\\\:B\end{array}\right]=\left[\begin{array}{c}U\\\:V\\\:W\end{array}\right]\left[\begin{array}{ccc}3.2406&\:-1.5372&\:-0.4986\\\:-0.9689&\:1.8758&\:0.4986\\\:0.0557&\:-0.2040&\:1.0570\end{array}\right]\left[\begin{array}{ccc}255&\:255&\:255\end{array}\right]\:\:$$

39 $$\:\left\{\begin{array}{c}V={(S}_{NGC}+16)/116\\\:U={A}_{c}/500+V\\\:W=V-{B}_{c}/200\end{array}\right.\:\:\:\:\:\:\:\:\:\:\:\:\:\:\:\:\:\:\:\:\:\:\:\:\:\:\:\:\:\:\:\:\:\:\:\:\:\:\:\:\:\:\:\:\:\:\:\:\:\:\:\:\:\:\:\:\:\:\:$$

**Fig. 3:**
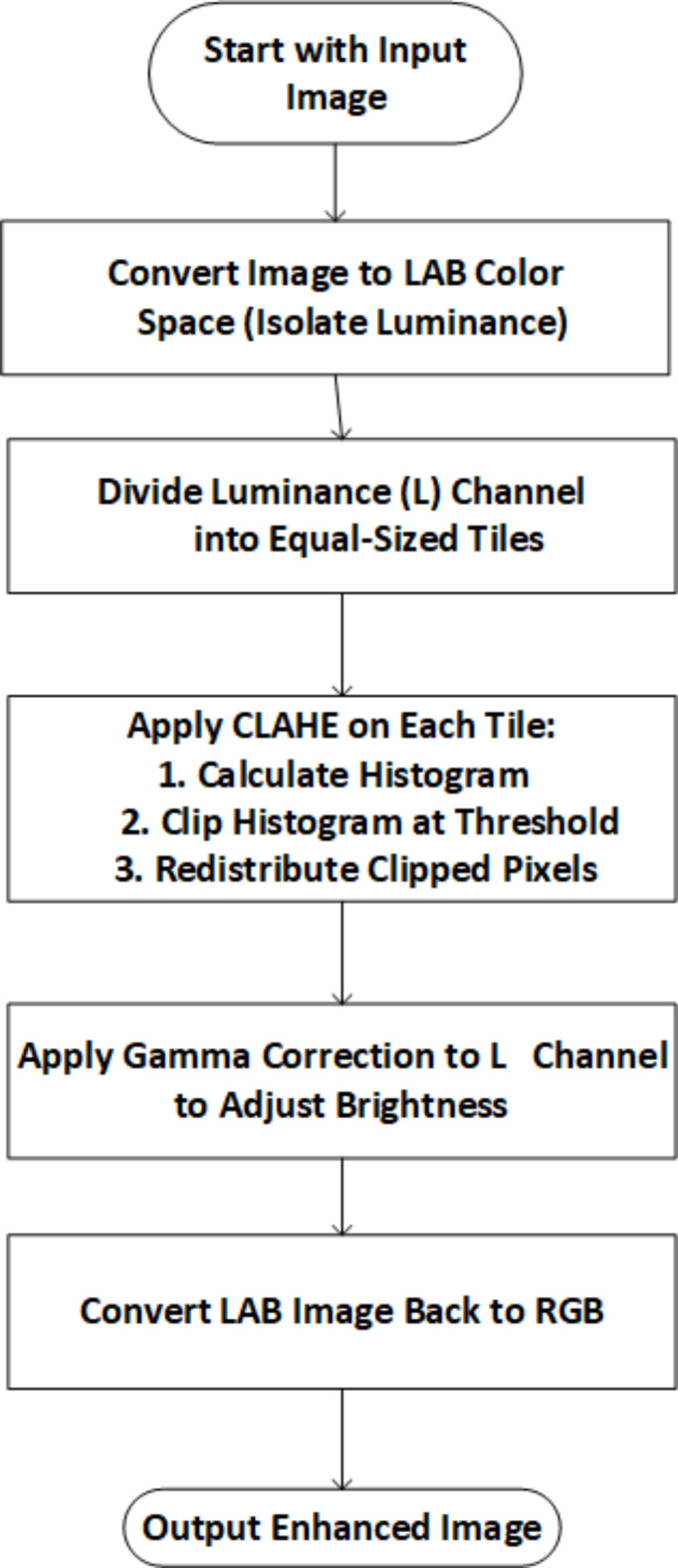
Flowchart for NGT-CLAHE.

In the lab color space, SNGC, AC, and BC are the reconstructed image component indices. Figure 3. represents the flowchart for NGT-CLAHE.

#### Adaptive thresholding

The adaptive thresholding is done using the improved adaptive Median Filter and IMF algorithms. The median returns a debased intensity value in noise detection methods when the processing window has more than 50% damaged pixels which is the median filter’s main problem. This approach uses valid pixels for window expansion and computes the median value to fix the median filter issue.

##### Step 1

Select a 3 × 3 window. If the processing pixel is wrong, Calculate,, and, which are the processing window’s median, minimum, and maximum respectively. Go to the following step if = - > 0 and = - > 0; otherwise, extend the window 2n + 1, (W + 2), repeat step 1.

##### Step 2

Determine the extended window D’s new ,, and .

If $\:{D}_{1}$=$\:{W}_{i,j}$-$\:{W}_{minimum}$ > 0 and $\:{D}_{2}$=$\:{W}_{i,j}$-$\:{W}_{maximum}$ > 0, keep the current processing pixel value; otherwise, replace it with the window B’s median $\:{W}_{mediam}$.

The pixels’ validity is tested as follows:

$\:{W}_{i,j}$ denotes the processing pixels. The maximum and minimum grey level noise intensity and starting size are a, b, and M respectively. The valid fleet window pixels are represented as b and intensity a.

**Fig. 4:**
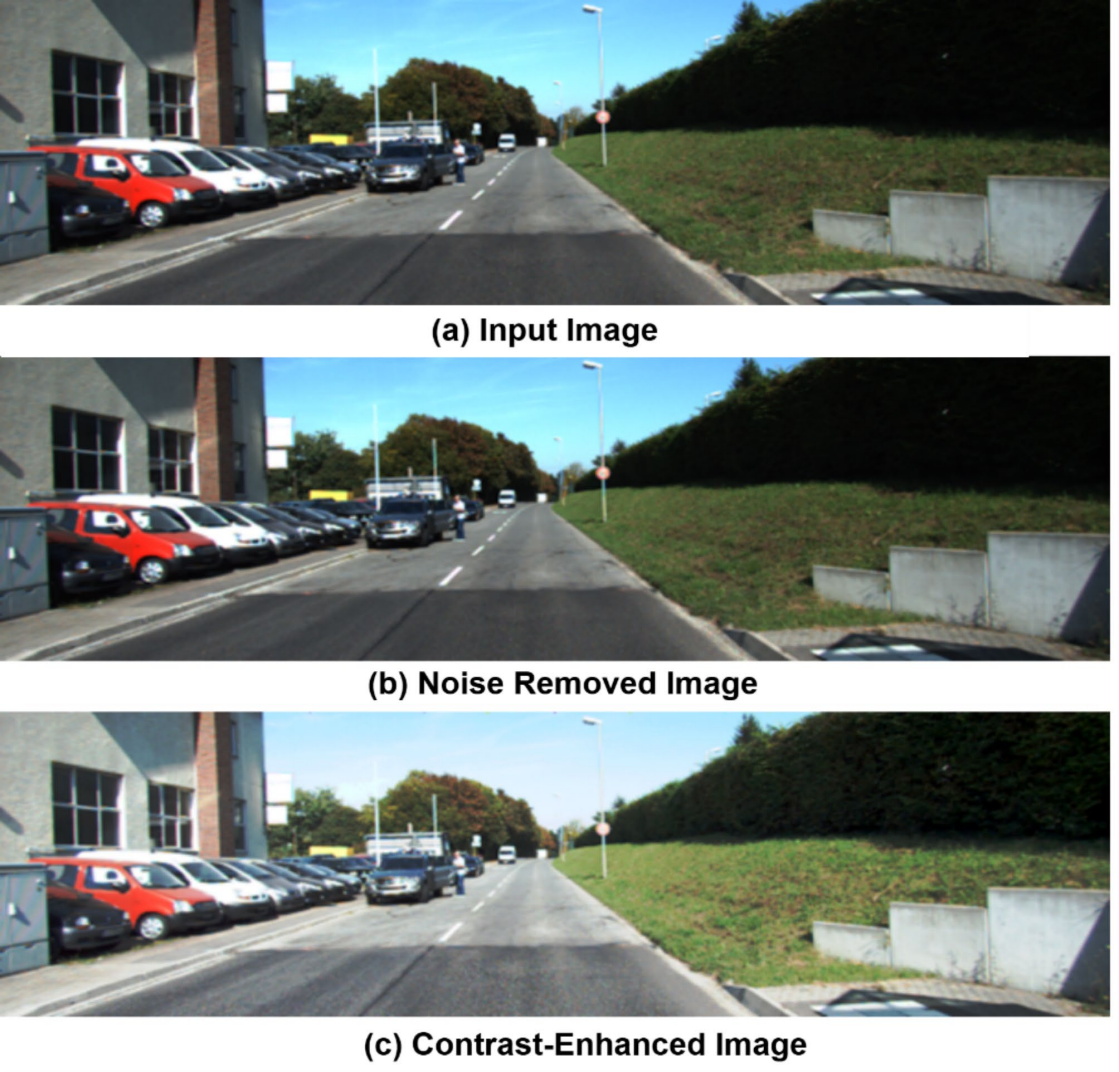
Image Enhancement Techniques. (**a**) Original Image, (**b**) Noise Removal using IAEKF, and (**c**) Contrast Enhancement using NGT-CLAHE.

Figure 4. illustrates the transformative impact of preprocessing techniques on image quality for autonomous driving applications. Initially, the input image suffers from noise and low contrast, hindering accurate object detection and scene understanding. However, upon applying noise removal techniques such as Improved Adaptive Extended Kalman Filter (IAEKF), the image undergoes significant improvement, exhibiting smoother textures and reduced visual noise. This enhancement enables more reliable object detection and tracking. Further refinement achieved through Contrast enhancement was done using a Normalised Gamma Transformation CLAHE (NGT-CLAHE), resulting in heightened contrast that accentuates critical features like road edges, lane markings, and objects. These preprocessing steps ensure accurate object detection, scene segmentation, and informed decision-making in autonomous driving systems. By ameliorating image quality, these techniques substantially contribute to autonomous vehicles’ overall safety, reliability, and performance, mitigating potential risks and failures.

### Orientation based multi segmentation

The segmentation structure in our proposed system employs a multi-stage approach to achieve comprehensive scene understanding from preprocessed images captured across various fields of view (FoV) and orientations. By integrating images from three distinct FoVs (90°−30°, 30°−30°, and 30°−95°), this block overcomes the limitations of single-FoV analysis, providing a holistic scene understanding. Each FoV undergoes tailored segmentation techniques: instance segmentation (90°−30°) accurately identifies individual objects and boundaries; panoptic segmentation (30°−30°) combines instance and semantic segmentation for detailed object and context representation; and semantic segmentation (30°−95°) classifies pixels into semantic categories (e.g., road, building, vehicle, pedestrian). The outputs are then fused using Light-G Net, a lightweight neural network optimized for real-time processing. By combining information from these segmentation approaches, Light-G Net generates a unified representation capturing fine-grained object details and high-level scene context, which is then passed to the next pipeline stage for multi-sensor fusion.

### Dense net based multi-image fusion

The DenseNet structure initiates with an input layer that processes segmented data after the preprocessing techniques to facilitate further analysis. It features an interconnected series of components designed to enhance feature extraction and improve performance in tasks like image classification and object detection. Following the input layer, a transition architecture effectively manages the flow of features through the network, reducing the dimensionality of feature maps to control model complexity and computational cost. This stage incorporates several vital operations, including batch normalization, which normalizes activations from the previous layer, stabilizes the learning process, and enhances convergence speed while minimizing the risk of overfitting. Subsequently, the Rectified Linear Unit (ReLU) activation function introduces non-linearity, enabling the network to learn complex patterns. We utilize a 1 × 1 convolutional layer to adjust the number of feature maps while preserving spatial dimensions, which acts as a bottleneck to reduce computational burdens and enhance feature interactions. Furthermore, average pooling (AvgPool) is employed to downsample feature maps, decreasing spatial dimensions and contributing to translation invariance.

**Fig. 5:**
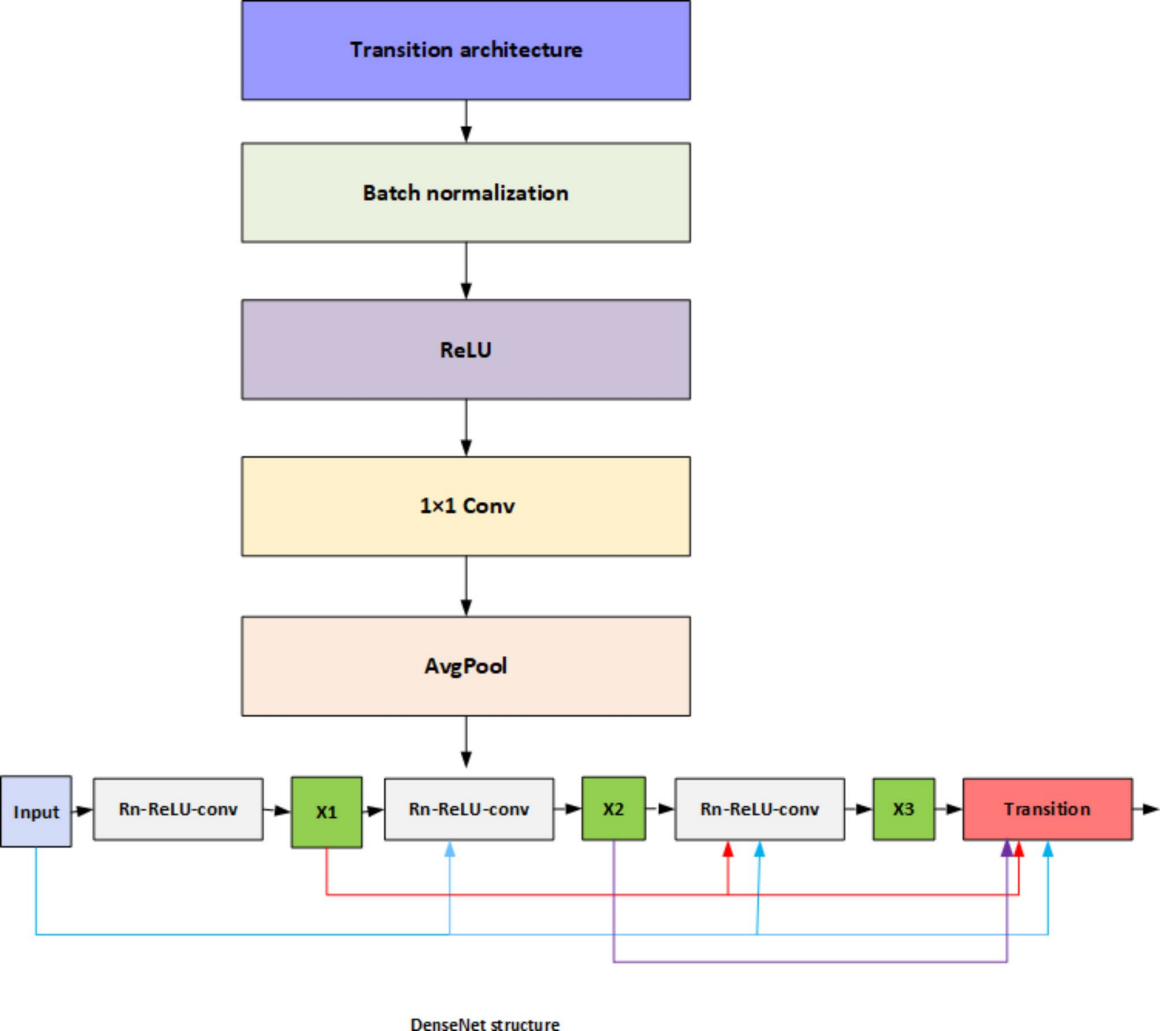
DenseNet structure.

Figure 5. shows the DenseNet structure, which consists of three Dense Blocks labeled X1, X2, and X3. These Dense Blocks, each with multiple layers of ReLU-activated convolutional layers densely connected, encourage feature reuse and enhance efficient representation learning. Transition layers between these dense blocks are essential for maintaining manageable dimensions, ensuring effective gradient propagation throughout the network, and mitigating the vanishing gradient problem associated with deeper architectures. The structure culminates in an output layer that caters to specific tasks like image classification or segmentation, enabling the model to generate predictions based on its acquired rich feature representations. The DenseNet structure skilfully combines dense connectivity, batch normalization, ReLU activation, 1 × 1 convolutions, and average pooling to make a solid and practical framework for processing complex data, which makes it useful for a wide range of computer vision applications.

**Fig. 6:**
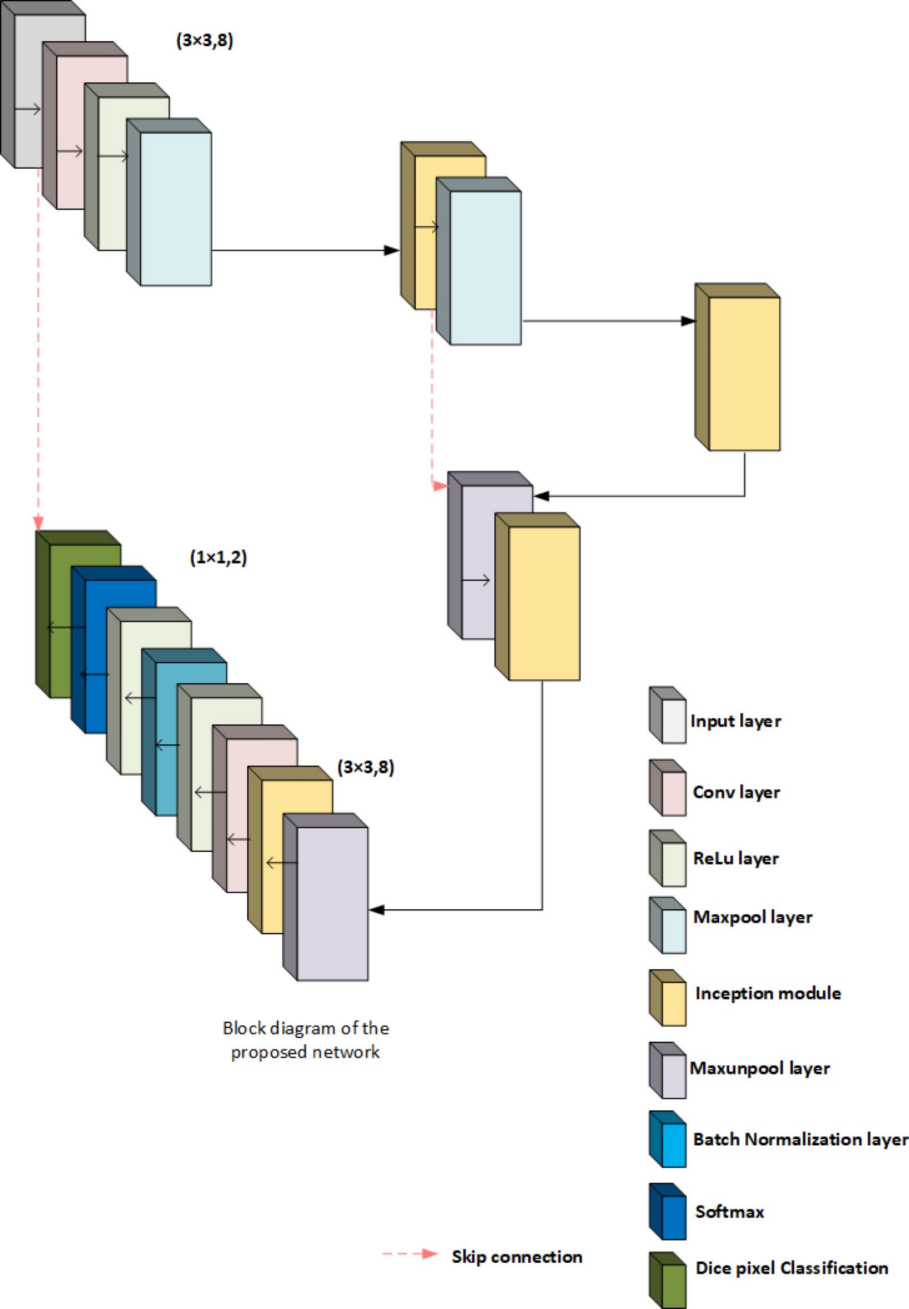
Block diagram of the proposed Network.

The proposed network is structured around an input image, and the initial data is fed into the system. This image leads to constructing a pixel-wise segmentation map. The process begins with convolutional layers that extract relevant features, followed by ReLU activation to introduce non-linearity. Max-pooling layers subsequently process feature maps to reduce spatial dimensions, ensuring computational efficiency while maintaining essential information. We integrate an Inception block after the first max-pooling layer, which enables multi-scale feature extraction and enhances the network’s capacity to capture complex patterns. The architecture incorporates encoding and decoding processes, with each Inception block as a bridge between these stages, as illustrated in Fig. 6. The decoder employs max-unpooling and additional inception blocks, followed by up-sampling layers to restore spatial information. After up-sampling, convolutional layers (CL), non-linear activations, and batch normalization layers (BN) refine the feature representation. The architecture culminates in Dice pixel classification following a softmax layer, enabling effective multi-class segmentation and producing a detailed pixel-wise classification output that indicates various objects, such as vehicles and pedestrians, within the segmented areas.

The design consists of four Inception blocks, with the initial one following a down-sampling operation. In each encoder block, the convolutional layers generate distinct features from the input feature maps, all of which are ReLU-activated. The feature maps are processed through 2 × 2 non-overlapping max-pooling or unpooling layers, each with a stride of 2, influencing the resolution and detail preserved throughout the network. The architecture’s motivation includes a deliberate reduction in pooling layers, as excessive pooling can lead to the loss of critical spatial information. To counter this, the network maintains a relatively small number of convolutional layers, optimizing convolutional filters to retain structural integrity. Moreover, skip connections bridge the encoder and decoder components, facilitating feature preservation throughout the learning process. This choice is driven by the notion that maintaining feature integrity within each convolutional layer can help mitigate the semantic gap on the encoder side, all while minimizing computing overhead during decoding. By strategically reducing pooling layers, the architecture is designed to retain fine-grained structures vital for tasks such as vehicle image segmentation. Integrating Inception blocks, max-pooling and unpooling layers, and skip connections contributes to a robust and efficient network capable of effective pixel-wise segmentation, ensuring high performance in complex image processing tasks.

The decision-making network, which uses grid mapping and the Energy Valley Optimizer (EVO) for autonomous path and lane selection, is shown in Fig. 1. By combining offline and online data, the grid map enables the car to traverse its surroundings adaptively while considering historical route data and current changes. By assessing the quality of picture segments and prioritizing pixels with the best visual quality, the EVO algorithm is used to improve route accuracy. This technique enhances decision-making reliability even in challenging situations by methodically evaluating and orienting the vehicle toward regions with high-quality visual input. This is further enhanced by YOLO V7, which can differentiate between moving and stationary objects to provide precise multi-object tracking—a crucial component of the vehicle’s safe and effective navigation from the starting point to the destination.

### Grid map-based path and lane selection

The decision-making system guides a self-driving car from its starting point to its destination to achieve the user’s aim. The decision-making system also considers the car’s state, the environment’s internal representation, traffic restrictions, passenger safety, and comfort.

#### Energy Valley optimizer (EVO)

In this study, we created the online grid maps by combining the offline and online maps with the **Energy valley optimizer** (EVO) method. In complicated situations with multi-object tracking, where effective decision-making is essential, especially under difficult driving circumstances, the DQL model’s setup works effectively. Stable learning is facilitated by the high experience buffer length and batch size, while the gradient and goal update frequency Power the Valley Optimizer (EVO) is used in autonomous driving to improve the accuracy of path and lane selection by improving grid mapping using both historical and real-time data. To prioritize routes with high-quality visual information to direct the vehicle’s trajectory, EVO assesses the quality of picture segments. In complicated contexts, where autonomous cars must accurately perceive their surroundings to make safe judgments, this is crucial. EVO optimizes for quality even under difficult circumstances by evaluating the visual quality of segments and modeling decay mechanisms that mimic movement toward segments with better visual input.

With its focus on picture quality instead of extensive environment modeling, EVO provides a more straightforward and adaptable method than conventional path optimization methods such as deep Q-learning networks (DQN) and other field-based models. While DQN techniques offer real-time flexibility by employing reinforcement learning to select choices based on cumulative reward metrics, EVO’s decay mechanisms and grid mapping concentrate on segment-based and visual prioritizing, which lowers computing overhead and improves real-time responsiveness. The EVO technique is particularly helpful in path-planning settings where dependable lane selection and rapid visual interpretation are more important than intricate learning frameworks. The steps are explained below.

Step 1: The initializing method assumes solution pixels (image patches) as picture segments with varied quality levels in the dataset, a specified region of the original image.

40 $$\:\:Y=\left[\begin{array}{c}{Y}_{1}\\\:{Y}_{2}\\\:\vdots\\\:{Y}_{j}\\\:\vdots\\\:{Y}_{n}\end{array}\right]=\left[\begin{array}{ccccc}{Y}_{1}^{1}{Y}_{1}^{2}&\:\dots\:&\:{Y}_{1}^{i}&\:\dots\:&\:{Y}_{1}^{e}\\\:{Y}_{2}^{1}{Y}_{2}^{2}&\:\dots\:&\:\vdots&\:\dots\:&\:\vdots\\\:\vdots&\:\dots\:&\:\ddots\:&\:\dots\:&\:\vdots\\\:{Y}_{j}^{1}{Y}_{j}^{2}&\:\dots\:&\:{Y}_{j}^{i}&\:\dots\:&\:{Y}_{j}^{e}\\\:\vdots&\:\dots\:&\:\ddots\:&\:\dots\:&\:\vdots\\\:{Y}_{n}^{1}{Y}_{n}^{2}&\:\dots\:&\:{Y}_{n}^{i}&\:\dots\:&\:{Y}_{n}^{e}\end{array}\right],\left\{\begin{array}{c}j=\text{1,2},.n\\\:i=\text{1,2},.e\end{array}\right.$$

41 $$\:{Y}_{j}^{i}={Y}_{j,min}^{i}+rand.\left({Y}_{j,max}^{i}-{Y}_{j,min}^{i}\right),\left\{\begin{array}{c}j=\text{1,2},.n.\\\:i=\text{1,2},.e.\end{array}\right.$$

The pixel count is n. The image dimension is considered along the width and height of the considered image. “The jth starting position choice variable is $\:{Y}_{j}^{i}$.$\:\:{Y}_{j,\:min}^{i},\:{Y}_{j,}^{i}$ max represents the lower and upper thresholds of the ith variable on the jth pixel and is a consistently circulated random number in the range[0,1].

Step2:The objective function evaluation for each image segment determines its image quality. These aspects are mathematically described as:

42 $$\:ET=\frac{{\sum\:}_{j=1}^{n}{IQ}_{j}}{n},j = 1,2,\dots{n}.$$

Step3-The objective function assessments determine the image segments’ quality level:

43 $$\:Q{L}_{j}=\frac{{IQ}_{j}-BQ}{WQ-BQ},j = 1,2,\dots{n}.$$

If $\:Q{L}_{j}$ stays the quality level of the jth pixels, BQ and WQ are the pixels with the greatest and worst quality levels in the original image, comparable towards the minimum and maximum objective function values obtained.

The quality level of a pixel is taken into consideration in the main exploration loop of the EVO if it is higher than the Enrichment Threshold ($\:{IQ}_{j}$ > ET), which illustrates decay using alpha, beta, or gamma schemes. In this case, the Quality Threshold (QT) of the dataset is imitated by generating a random number in the interval [0, 1]. Alpha and gamma decay are assumed to occur if a particle’s Quality level exceeds the Quality Threshold ($\:Q{L}_{j}$ > ET), as these two decays are likely for weightier pixels with greater quality levels. Because beta decay occurs in additional unbalanced image segments by lower quality levels, it is assumed to occur when an image segment’s quality level is lower than the Quality Threshold ($\:Q{L}_{j}\le\:QT$).In this case, the image segment goes through a process of updating its positions where a controlled movement is made toward the segment with the best quality level ($\:{Y}_{QL}$) and the center of the segment ($\:{Y}_{CS}$). These algorithmic features imitate the image segment’s propensity to approach the Quality Range, where the majority of known segments are located and the majority of them have higher degrees of quality. These elements are expressed mathematically as follows:

44 $$\:{Y}_{CS}=\frac{{\sum\:}_{j=1}^{n}{Y}_{j}}{n},j=\text{1,2},.n.\:$$

45 $$\:{Y}_{j}^{New1}={Y}_{j}+\frac{\left({d}_{1}\times\:{Y}_{QL}-{d}_{2}\times\:{Y}_{CS}\right)}{Q{L}_{j}}$$

where $\:{Y}_{j}^{New1}$and $\:{Y}_{j}$ are the anticipated and current position vectors of *jth*$\:{Y}_{j}$ pixels in the image space, $\:{Y}_{QL}$is the position vector of the pixel with the best quality level,$\:\:{Y}_{CS}$ is the position vector for the center of the pixel, $\:{Y}_{QL}$ is the quality level of the *jth* particle, $\:{d}_{1}$ and $\:{d}_{2}$ are two random numbers in the range of [0, 1] which determines the amount of pixels’ movement.

Another position-updating process for particles using beta decay involves controlled movement toward the pixel with the best quality level ($\:{Y}_{QL}$) and a neighboring pixel ($\:{Y}_{NP}$) without affecting the pixel’s quality level. These are mathematically expressed:

46 $$\:{Y}_{j}^{New2}={Y}_{j}+\left({d}_{3}\times\:{Y}_{QL}-{d}_{4}\times\:{Y}_{NP}\right),j=\text{1,2},.n.$$

where $\:{Y}_{j}^{New2}$ and$\:{\:Y}_{j}$ are the anticipated and current position vectors of the jth pixel in the image space,$\:\:{Y}_{QL}$ is the best quality pixel, $\:{Y}_{NP}$is the neighboring particle around the jth pixel, $\:{d}_{3}$and $\:{d}_{4}$ are two random numbers in the range [0, 1] that determine pixel movement. Pixel is below the enrichment Threshold ($\:{IQ}_{j}\le\:ET$), for such moves and, the random image space movement is determined:

47 $$\:{Y}_{j}^{New}={Y}_{j}+d,j=\text{1,2},.n.$$

Where $\:{Y}_{j}^{New}\:$and $\:{Y}_{j}$are the incoming and present position vectors of the jth pixels in the image space, and d is a random value between 0 and 1 that governs the pixel movement. If the pixel’s enrichment level is higher than the enrichment threshold two new position vectors, $\:{Y}_{j}^{New1}$and$\:{Y}_{j}^{New2}$, are generated at the end of the EVO’s main loop. If it is lower, only one is generated. At each state, the freshly created vectors are merged with the current dataset.

**Figure:**
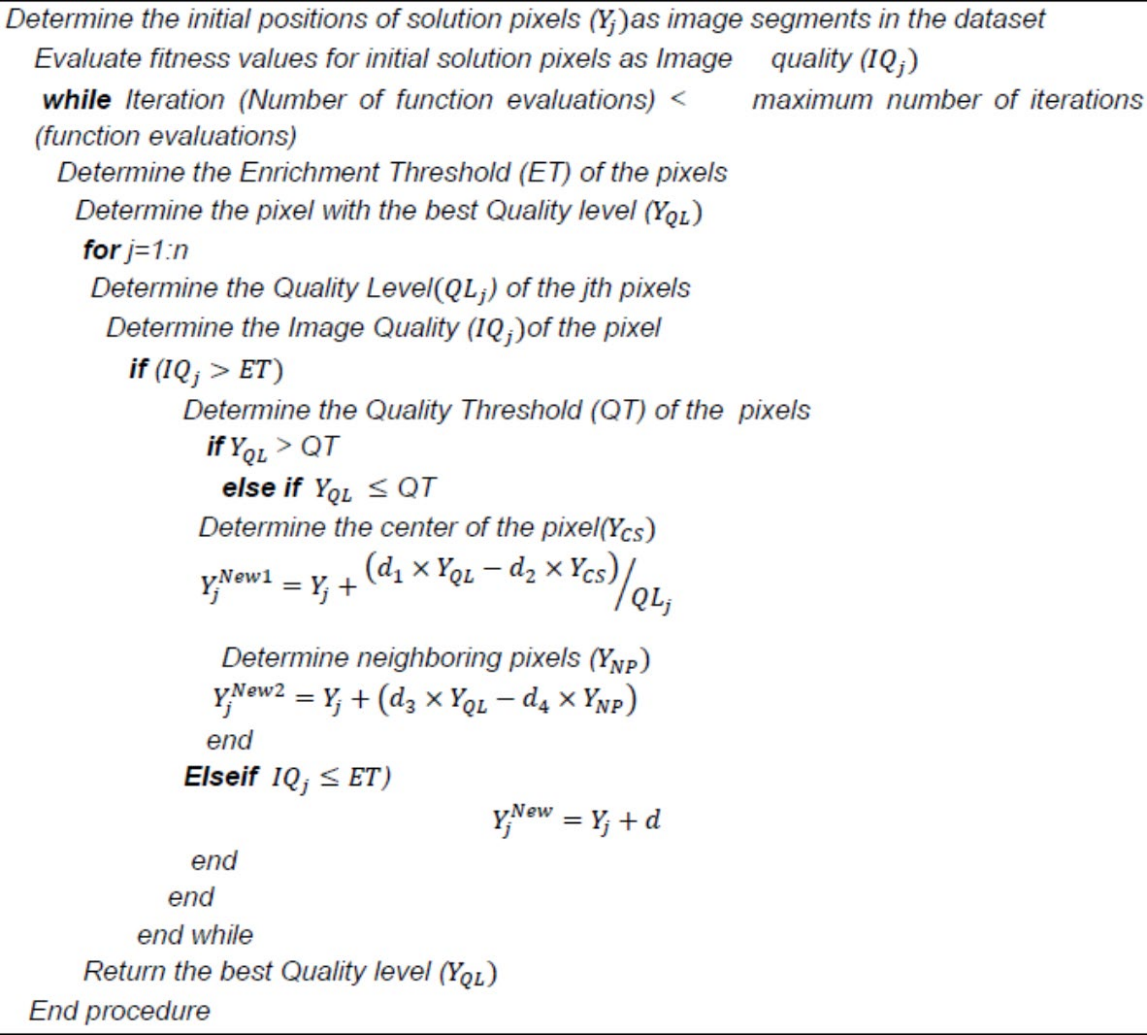
**Algorithm 1**. Energy Valley Optimizer (EVO).

#### YOLOV7

**Fig. 7:**
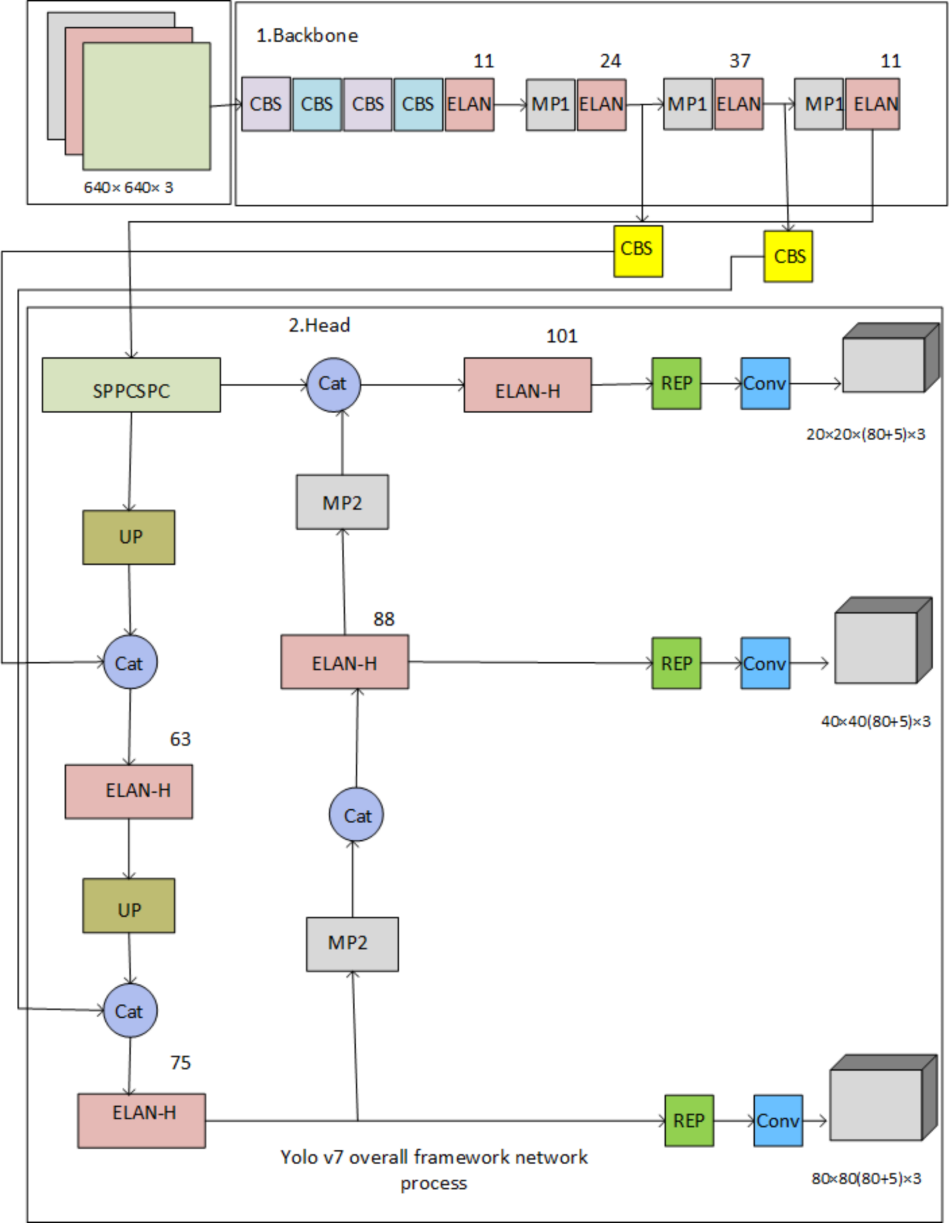
YOLOv7 overall framework network process.

The classification of moving and unmoving objects for accurate path selection is conducted using the YOLO V7 model, as illustrated in Fig. 7. This framework is a critical component for autonomous navigation by enabling real-time recognition and differentiation between stationary and moving objects. YOLO V7, short for “You Only Look Once,” is an advanced deep-learning model designed for rapid object detection. It operates by dividing an input image into a grid, with each cell estimating bounding boxes and class probabilities for any objects present. Aimed at high-speed processing, YOLO V7 is well-suited for real-time applications, such as autonomous driving, where it can swiftly and accurately identify objects, even in dynamic environments. Upon recognizing and classifying objects, the model provides the vehicle with essential real-time data regarding obstacles and various route options, facilitating better path selection. This capability enables the autonomous system to safely navigate, avoid obstacles, and adapt to changing conditions by categorizing objects into moving and stationary groups.

Before entering the backbone network, images are scaled to 640 × 640 pixels. The head layer network generates three feature map layers of varying sizes, and the Rep and conv output represents the prediction results. With three anchor boxes, the model outputs (x, y, width, height, object score) for each anchor culminate in a total output size calculated as (80 + 5) × 3 = 255, corresponding to the size of the feature map.

The classification of moving and unmoving objects for accurate path selection is performed using the YOLO V7 model. The Yolov7 framework network process is depicted in Fig. 7. For autonomous cars to navigate accurately and adaptively, the YOLO V7 model is used in this work as a key element for path selection by recognizing and differentiating between moving and stationary objects in real-time. YOLO V7, or “You Only Look Once,” is a sophisticated deep-learning model that is tuned for quick item recognition. It splits photos into a grid and uses each cell to estimate bounding boxes and class probabilities for the things it includes. Because it prioritizes speedy processing, YOLO V7 is ideal for real-time applications like autonomous driving, as it can identify objects rapidly and accurately, even in dynamic surroundings. After identifying and classifying things, the model helps the vehicle make decisions about which path to take by giving it real-time information about nearby impediments and other routes. The autonomous system can safely and effectively plan routes, avoid impediments, and react to changing conditions thanks to this division of objects into moving and stationary categories.

Before entering the backbone network, the image is scaled to 640 × 640 pixels. The head layer network generates three feature map layers of different sizes, and the Rep and conv output represents the prediction results. Coco’s dataset has three anchors and outputs (x, y, w, h, o) that coordinate locations and backdrops before and after 80 output categories. Each layer’s output is (80 + 5) × 3 = 255 multiplied by the feature map’s size.

#### Deep Q Network (DQN)

In standard Q-learning, the Q-table can hold the Q value of each state-action combination when the state and action space are discrete and the dimension is modest. Our autonomous navigation system employs a Deep Q-Network (DQN) architecture to learn optimal path selection strategies. The DQN comprises three main pathways: a state pathway, an action pathway, and a common pathway.

***State pathway***: This pathway processes the environmental state input, which comprises four components: the current image data processed by the multi-sensor fusion module (described in Sect. 3.3). The state data initially passes through a preprocessing layer. The pre-processed input is fed into two fully connected layers containing 24 neurons. A ReLU activation function is applied after the first fully connected layer to introduce non-linearity.

##### Action pathway

This path processes an action input in one dimension. An image input layer handles the action data before being sent via a fully linked layer with 24 neurons.

##### Common pathway

An addition layer merges the outputs from the state and action routes. This combined output then produces the Q-value for the specified state-action combination after it passes through a second ReLU layer and, lastly, a fully connected layer with a single neuron.

***DQN parameters and Training***: To ensure steady and effective training, the DQL model is adjusted using the following parameter settings: Learning Rate, Gradient Threshold, DQN Agent Options: UseDoubleDQN: false, Target Update Method: periodic, Target Update Frequency, Experience Buffer Length, Discount Factor (γ), and Mini-batch Size.

The DQN was trained for 100 epochs. The training process was monitored using several metrics, including the average reward per episode and the mean squared error. These metrics are briefly presented in Sect. 4.

The Q-Table is unrealistic when image pixels are the input. The Q-Table updating is usually turned into a function fitting problem, and related states get alike output actions. The DNNs automatically extract complicated characteristics. CNN and Q-Learning approximates the Q function. The network has two full connection layers (fc1 has 12500 nodes; fc2 has two nodes) and two convolution layers (conv1 is twisted with 10 kernels of 2 × 2 with stride 1 and conv2 is convoluted with 20 kernels).

The algorithm obtained DQN training samples from experience replay and modified system parameters by an all-purpose Stochastic Gradient Descent (SGD) and backpropagation algorithms.

**Figure:**
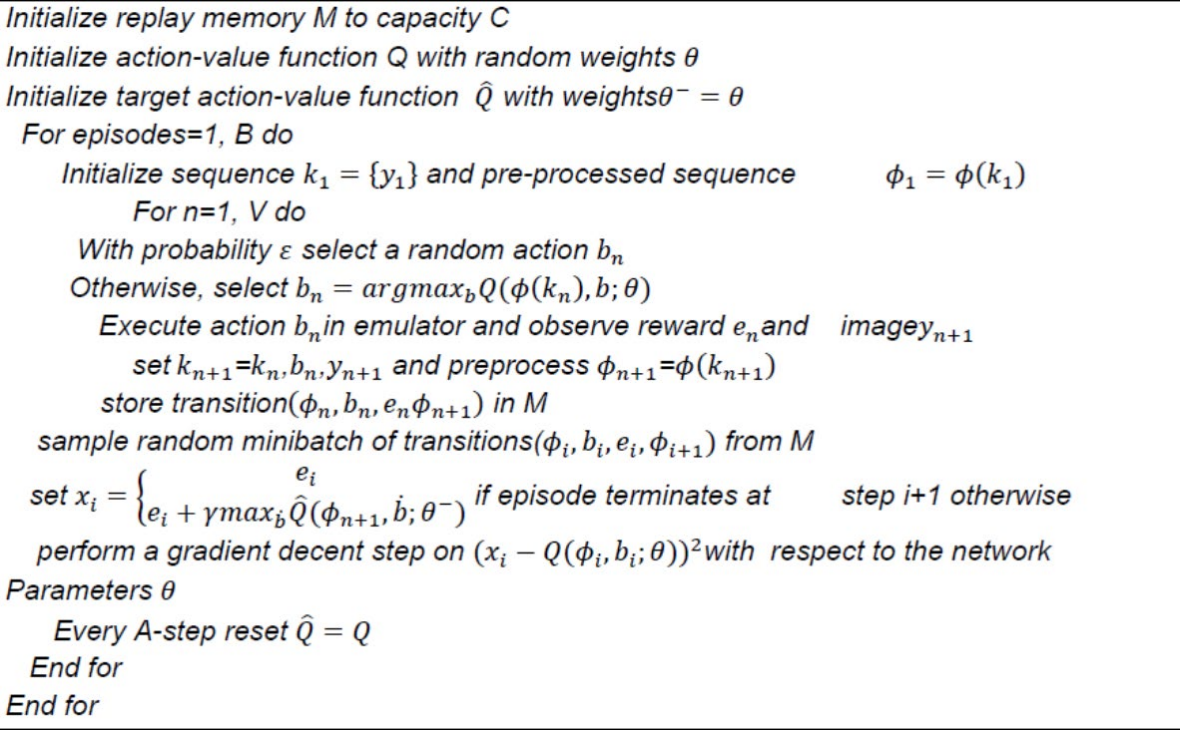
**Algorithm 2**. Deep Q-learning with experience replay.

- Training.

We produced initial Q values for each training image and saved them in a folder called “Targets”. The randomly initialized model output Q values were determined. Next, the model was fed a randomly selected batch of images from the training data. To estimate the action-value function, we used a Q-network, a neural network function$\:Q\left(k,b;\theta\:\right)\approx\:{Q}^{*}\left(k,b\right)$approximate with weight$\:\theta\:$. The model weight is adjusted based on changing loss functions H($\:\theta\:$)at each iteration.

48 $$\:\text{H}\left(\theta\:\right)\hspace{0.17em}=\hspace{0.17em}\text{F}\left[{\left(Target-Q(k,b;\theta\:)\right)}^{2}\right]$$

49 $$\:Target=e(k,b)+\gamma\:\text{m}\text{a}\text{x}\left({Q}_{1}\left({k}_{1},b\right)\right)$$

The $\:\gamma\:$max is selected as the current condition between the two activities. $\:{Q}_{1\:}$represents the Q values in the ‘Targets’ file. $\:{Q}_{1}$values were reorganized and stored in the same folder as the outputs from the trained model after a few epochs of training. The updated $\:{Q}_{1}$ results were utilized to train the model afresh. Several procedures were followed to generate the goal Q value and train the model to achieve the desired attributes.

#### Path selection methodology

The framework combines YOLO V7’s object detection capabilities with Deep Q-Network’s (DQN) decision-making abilities to enable autonomous cars to select optimal routes dynamically. This integration begins with YOLO V7 processing real-time sensor data to recognize and categorize surrounding objects, providing bounding boxes, object classes, and positions. The scene is divided into a grid for item identification, distinguishing between immovable objects and moving objects. The YOLO V7 detections are then transformed into a DQN-appropriate state representation, incorporating item categories, distances from the vehicle, current speed, and spatial locations. The DQN assesses potential courses of action based on this state information, determining a Q-value for each action. Rewards are assigned based on safety, speed, and path optimization. The action with the highest Q-value is selected, ensuring safe and effective path selection. The autonomous car executes the chosen action, adjusting course or speed to navigate identified obstacles. YOLO V7 continually updates the DQN status in real time, enabling rapid adaptation to change conditions. Reinforcement learning techniques can improve the DQN’s policy to enhance performance, balancing short-term actions with long-term path optimization. YOLO V7 can also be fine-tuned to maintain object detection speed and accuracy in various conditions. This integrated system dynamically adjusts routes to ensure efficiency and safety in challenging situations.

Our evaluation is based on comprehensive performance measures, assessing the model’s effectiveness across critical components in practical self-driving applications under harsh weather conditions. We optimize overall efficacy by coordinating pipeline stages, including segmentation, image fusion, tracking, noise reduction, and contrast enhancement. Comparative analyses with existing approaches are conducted using metrics such as accuracy, velocity-distance relation, and success rate, emphasizing overall system reliability. The YOLO V7 model was implemented on a hardware platform comprising an Intel Core i5-4590 S CPU @ 3.00 GHz, NVIDIA GeForce RTX 3080 GPU, 8GB RAM, and 1 TB HDD. The software configuration included Ubuntu 20.04 LTS OS, PyTorch 1.9, OpenCV 4.5, and MATLAB R2020a for data processing and visualization.

## Experimental results

This section includes an experimental analysis of the proposed DQN method for comparison with evaluation metrics. The results show that the proposed DQN achieves high efficiency. This section consists of four sub-sections: dataset, simulation setup, comparative analysis, and research summary.

### Dataset description

The KITTI dataset^52^ should include sophisticated object interactions like individuals using umbrellas or automobiles responding to slippery roads to reflect extreme weather conditions. The dataset accurately evaluates multi-object tracking systems under demanding situations by annotating ground truth trajectories, locations, and item behaviors affected by the weather. To establish a full evaluation platform, the dataset can be augmented with different road types, traffic volumes, and weather intensities. This enhanced KITTI dataset helps researchers and developers develop self-driving technology that functions well in bad weather, making autonomous vehicles safer and more capable. The KITTI dataset, a well-known standard for autonomous driving research, was used to evaluate the suggested approach. The KITTI collection contains a wide variety of sensor data, including LiDAR and camera pictures, as well as information for actual driving situations. It is extremely important for testing real-world applications since it includes annotated data for object identification, tracking, and path planning in various contexts, including traffic conditions, weather fluctuations, and urban and rural landscapes. With object labels for tasks like car, pedestrian, and cyclist recognition, as well as trajectories in various circumstances that mimic the complexity of real-world driving, the dataset comprises 7481 training photos and 7518 test images.

### Simulation Setup

The proposed Multi-Object Tracking using the DQN model is implemented and simulated using the MATLAB R2020a tool (https://www.mathworks.com/products/new_products/release2020a.html). To obtain successful simulation results, we tuned the system configurations in terms of hardware and software configurations respectively. The software configuration was tuned with a Windows 10 operating System (OS), a Central Processing Unit (CPU) processor of Intel(R) Core (TM) i5- 4590 S CPU @ 3.00 GHz, and a simulation tool of MATLAB with version R2020a. The hardware configurations were a hard disk and a RAM range of 1 TB and 8GB respectively.

### Comparative analysis

The study benchmarks its proposed Deep Q-Network (DQN)-based multi-sensor fusion and segmentation method against existing techniques like Memory-Augmented Neural Networks (MANN)^23^, CARL-D^18^, and Multi-Sensor Fusion-based Object Tracking and Association (MFOTA)^45^. The comparison uses several key performance metrics to evaluate the effectiveness and efficiency of each approach under various conditions.

#### Accuracy

We employ accuracy as a key performance metric to assess the effectiveness of our proposed image quality enhancement technique. The accuracy measures the percentage of correctly detected objects from all observed items, reflecting detection reliability in autonomous systems. High accuracy ensures precise obstacle recognition and safe navigation. It is represented as

50 $$\:Accuracy=\frac{TP+TN}{TP+TN+FP+FN}\times\:100$$

Where:

TP: True Positives (correctly identified vehicles).

TN: True Negatives (correctly identified non-vehicles).

FP: False Positives (non-vehicles incorrectly identified as vehicles).

FN: False Negatives (vehicles incorrectly identified as non-vehicles).

**Fig. 8:**
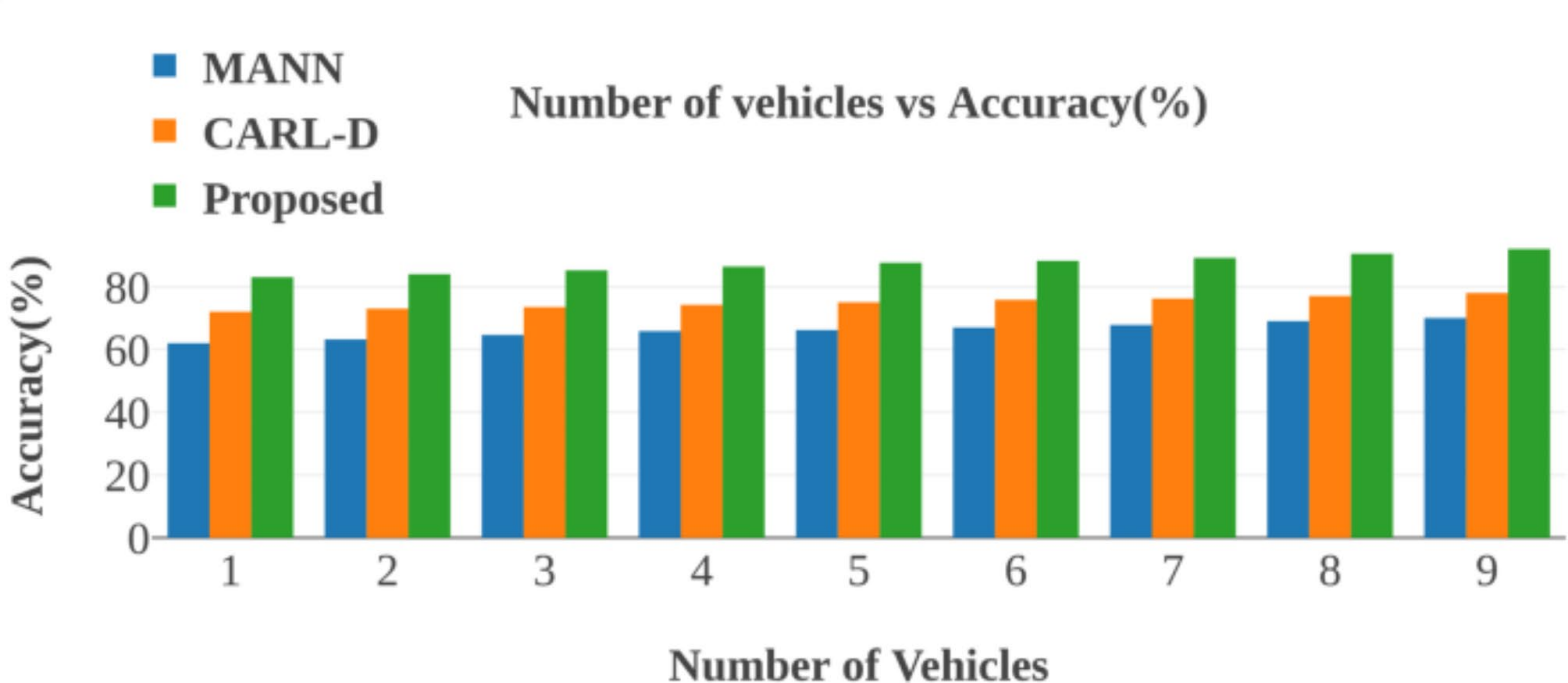
Accuracy of Vehicle Detection Across Varying Vehicle Counts.

The comparative graphical accuracy analysis in Fig. 8. highlights the enhanced effectiveness of the suggested strategy in precisely monitoring numerous vehicles as their number grows. When tracking nine cars, the proposed method achieves an accuracy of 92%, proving that it can manage intricate scenarios without sacrificing precision. By contrast, the MANN approach only reaches 70% accuracy, while CARL-D reaches 78% under the same circumstances, suggesting that both approaches noticeably lose accuracy as the number of vehicles increases. This decline in performance indicates that MANN and CARL-D may be unable to handle more complex scenes, possibly due to less effective tracking or sensor fusion algorithms. The suggested approach outperforms these current approaches by 11% on average, demonstrating its robust design and sophisticated sensor fusion, segmentation, and picture preprocessing capabilities. This overall improvement in performance suggests that the proposed approach is a more dependable and scalable way to track many objects in self-driving applications, even in difficult situations with many cars.

#### Velocity

Velocity, in this context, measures the speed at which the algorithm can process and track moving objects. The formula used is:

51 $$\:V\:=\:\:\frac{D}{n1-n0}\:\:\:\:\:\:\:\:\:\:\:\:\:\:\:\:\:\:\:\:\:\:\:\:\:\:\:\:\:\:\:\:\:\:\:\:\:\:\:\:\:\:\:\:\:\:\:\:\:\:\:\:\:\:\:\:\:\:\:\:\:\:\:\:\:\:\:\:\:\:\:\:\:\:\:\:\:$$

Where:

- Distance is the distance between two sensor readings.
- n1 - n0 represents the time difference between the two sensor readings.

Higher velocity suggests faster processing and reaction times, which are crucial for real-time applications. The DQN method demonstrates notably higher velocities than other approaches, particularly as the distance to the tracked objects increases. This superior speed is directly linked to improved real-time responsiveness.

**Fig. 9:**
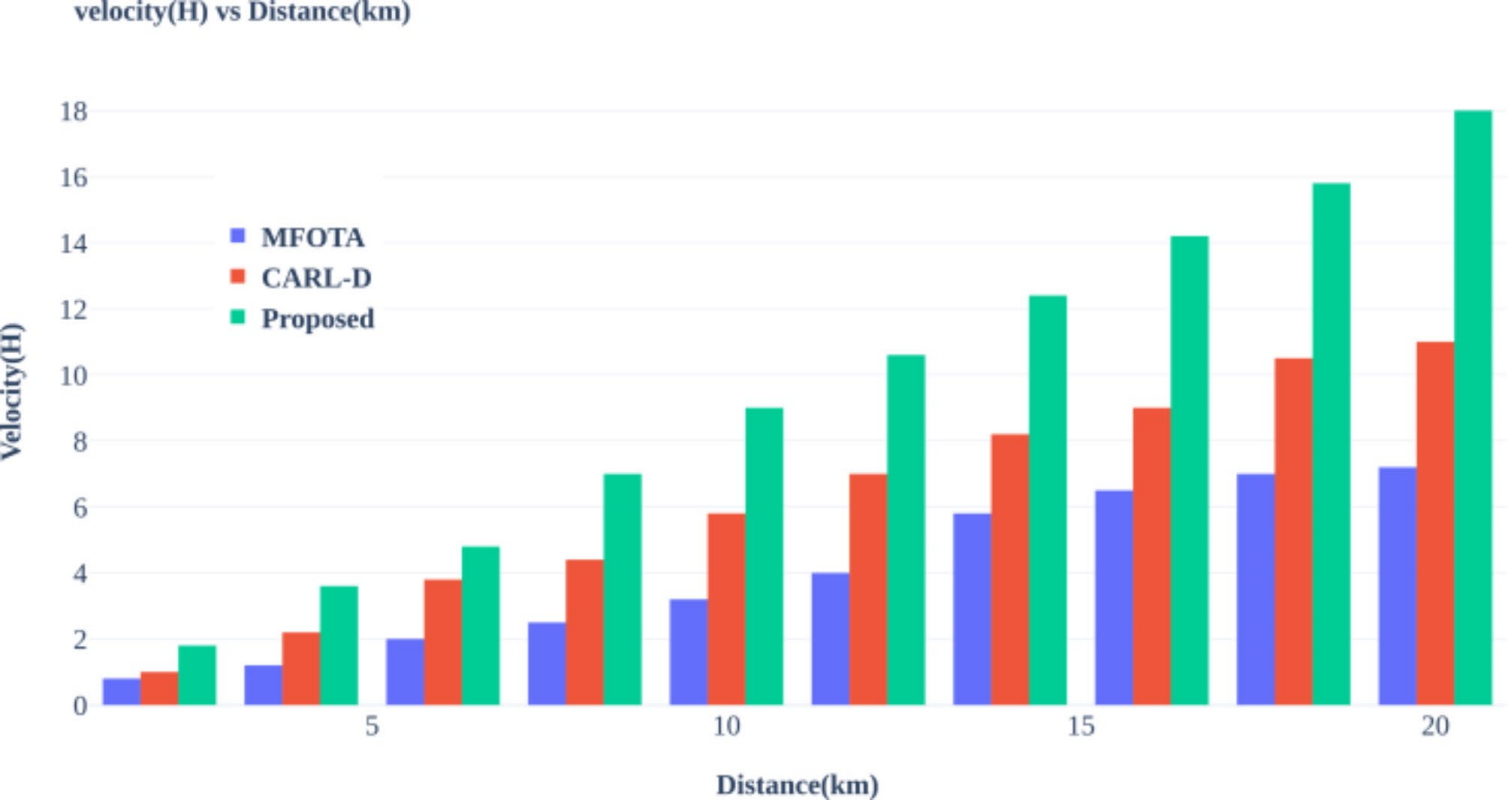
Performance Comparison of Object Tracking Methods: Velocity vs. Distance.

The velocity analysis findings in Fig. 9. show how well the suggested method performs compared to the current techniques, particularly MFOTA and CARL-D. The proposed approach achieves a velocity of 12 (H) at an initial distance of 5 km, while MFOTA and CARL-D only manage 5 (H) and 8 (H), respectively. When the distance reaches 20 km, the suggested approach stands out even more, achieving a velocity of 18 (H), which is noticeably faster than MFOTA’s 9.5 (H) and CARL-D’s 7.8 (H). While MFOTA and CARL-D only average 6.5 (H) and 5 (H), respectively, the suggested technique averages 7 (H). Compared to the current approaches, this continuously more incredible velocity shows that the proposed approach is more effective at dynamic object tracking, allowing quicker and more dependable real-time reactions.

#### Accuracy rate

Safe navigation and collision avoidance require balancing prolonged iterations for accuracy with real-time decision-making. To achieve this, the accuracy rate is crucial. Accuracy rate assesses the percentage of pixels correctly classified during image segmentation.

52 $$\:{AR}_{a}={\sum\:}_{b}{M}_{ab}$$

Where: Mab is the number of pixels predicted to be in class ‘b’ while belonging to class ‘a’.”

A higher accuracy rate indicates more precise segmentation. The DQN method exhibits a superior accuracy rate over other methods across various iteration counts, underscoring its ability to maintain high accuracy even with prolonged processing. The consistent, high accuracy rate emphasizes that the process produces high-quality segmentation results efficiently.

**Fig. 10:**
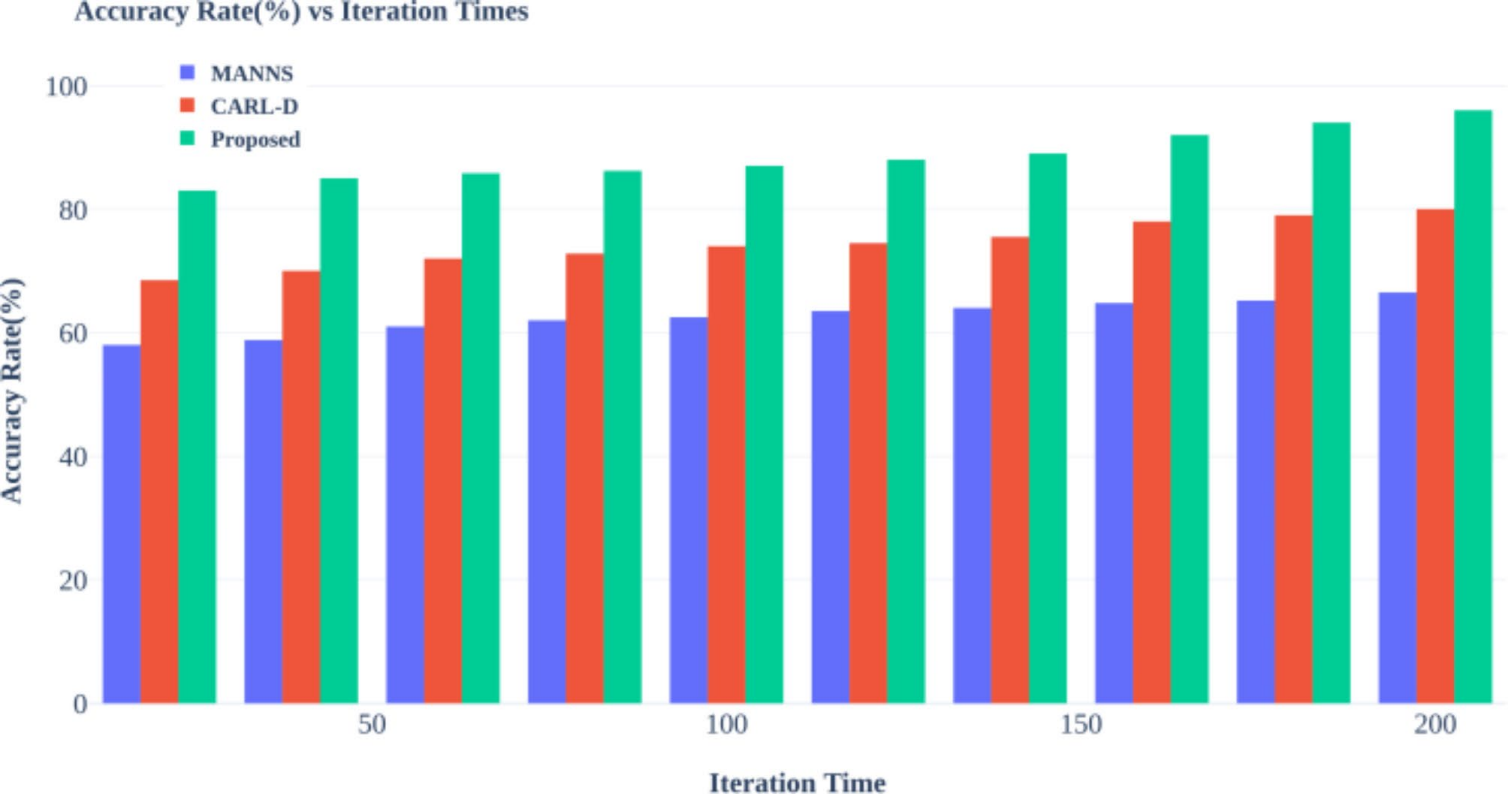
Performance Comparison of Object Tracking Methods: Accuracy Rate vs. Iteration Time.

Figure 10. graphically compares the accuracy rates for various approaches over increasing repetition periods. Even as iteration times grow, the suggested DQN technique maintains its efficiency and consistently obtains the best accuracy rate. The DQN method first achieves a 94% accuracy rate at 100 iterations, while CARL-D and MANN only attain 75% and 62%, respectively. Following 200 iterations, the DQN method achieves a 97% accuracy rate, whereas MANN and CARL-D only slightly increase to 78% and 65%, respectively. This shows that, in contrast to other techniques like MANN and CARL-D, the DQN approach not only offers a higher accuracy rate but also maintains its performance over time more successfully. The DQN technique is more dependable for lengthy, iterative activities in dynamic contexts because of its greater and more consistent accuracy rate. The DQN technique is more dependable for lengthy, iterative activities in dynamic contexts because of its more remarkable and consistent accuracy rate.

#### Success rate

Success rate measures the algorithm’s ability to track objects, balancing speed and accuracy successfully. It’s influenced by a speed-accuracy trade-off represented by a constant ‘V’ in the following relationship:

53 $$\:Speed=\raisebox{1ex}{$V$}\!\left/\:\!\raisebox{-1ex}{$SR$}\right.\:\:\:\:\:\:\:\:\:\:\:\:\:\:\:\:\:\:\:\:\:\:\:\:\:\:\:\:\:\:\:\:\:\:\:\:\:\:\:\:\:\:\:\:\:\:\:\:\:\:\:\:\:\:\:\:\:\:\:\:\:\:\:\:\:\:\:\:\:\:$$

The DQN method shows high and consistent success rates across different speeds. Its success rate maintains a high value, even at higher speeds, surpassing the other methods. This demonstrates a superior ability to track objects accurately and rapidly in dynamic scenes.

**Fig. 11:**
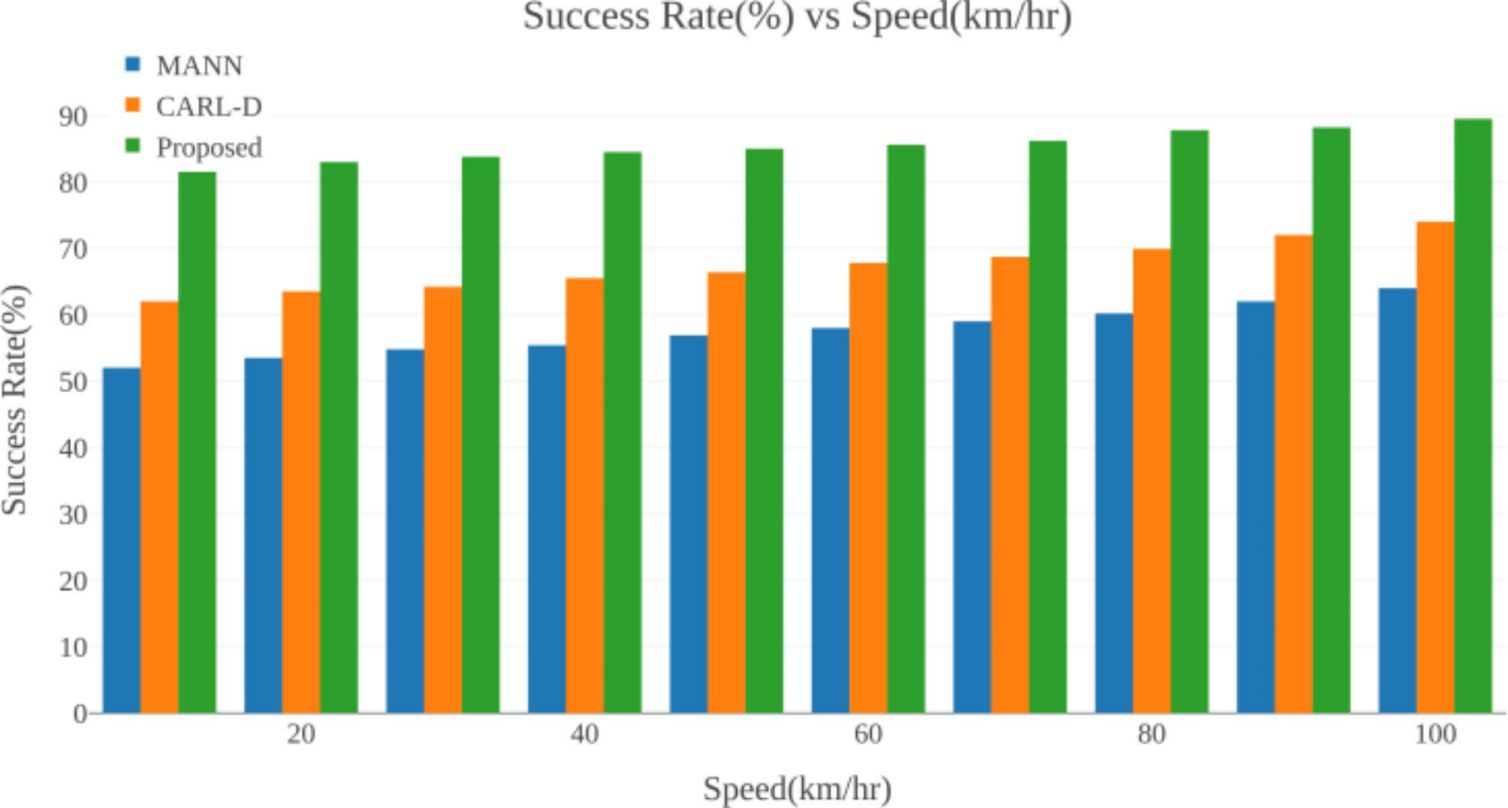
Performance Comparison of Object Tracking Methods: Success Rate vs. Speed.

The comparison of success rates for various approaches at different speeds is shown in Fig. 11. The suggested DQN approach has an initial success rate of 82% at 10 km/h, although the MANN and CARL-D approaches have higher initial success rates of 84% and 85.6%, respectively. The DQN strategy, however, performs noticeably better than the other strategies as speed rises. The DQN approach has an 89% success rate at the highest tested speed of 100 km/h, while MANN and CARL-D only achieve 74% and 65% success rates, respectively. The DQN approach has an average success rate of 86.8%, compared to 57.6% and 68.4% for MANN and CARL-D, respectively. These findings show that, in contrast to alternative strategies, the DQN technique is more reliable and efficient for dynamic applications as it retains a high and steady success rate even at faster rates.

#### Success ratio

The success ratio reflects the relationship between detection thresholds and the algorithm’s success in correctly identifying objects. The equation is given as:

54 $$\:Success\:Ratio=T\left(threshold\right)\:\:$$

Where ‘T’ represents a function that maps the detection threshold to the success ratio.

Fine-tuning of detection thresholds impacts the balance between accuracy and the number of false positives. The results indicate that the DQN-based method maintains a high success ratio across a range of thresholds.

**Fig. 12:**
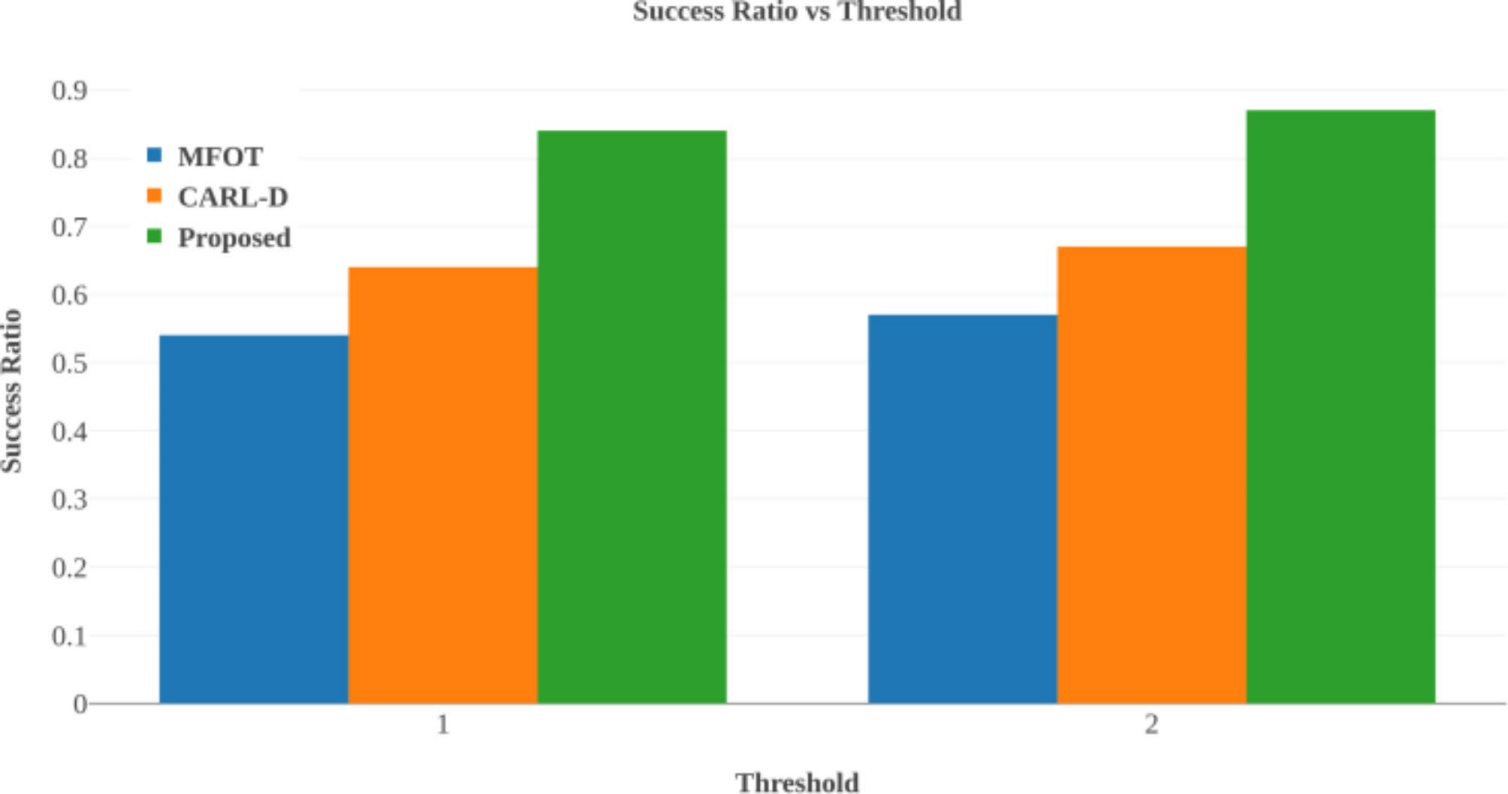
Performance Comparison of Object Tracking Methods: Success Ratio vs. Threshold.

Figure 12. displays a graphical comparison of success ratios for various approaches across threshold levels. The DQN methodology consistently outperforms other approaches like MFOTA and CARL-D, demonstrating efficiency. The DQN approach produces a success ratio of 0.85 at the first threshold level. This success ratio rises to 0.88 at the second threshold, proving its resilience under various circumstances. On the other hand, MFOTA attains a success ratio of 0.57 at the second threshold, while CARL-D only manages to reach 0.68. These findings demonstrate how well the DQN approach maintains a high success ratio across thresholds, which makes it a more dependable option for applications that need constant performance over a range of operating thresholds.

#### Mean squared error

MSE quantifies the average squared difference between predicted and actual values, measuring the error in predictions. The study utilizes a constant to balance MSE, and processing time is given as

55 $$\:C=C\text{*}T\:\:\:\:\:\:\:\:\:\:\:\:\:\:\:\:\:\:\:\:\:\:\:\:\:\:\:\:\:\:\:\:\:\:\:\:\:\:\:\:\:\:\:\:\:\:\:\:\:\:\:\:\:\:\:\:\:\:\:\:\:\:\:\:\:\:\:\:\:\:\:\:\:\:\:\:\:\:\:\:\:\:\:$$

where C = MSE, and T = processing time.

Lower MSE indicates better predictive performance. The DQN method consistently demonstrates lower MSE values across varying time periods, outperforming competing approaches in terms of predictive accuracy.

**Fig. 13:**
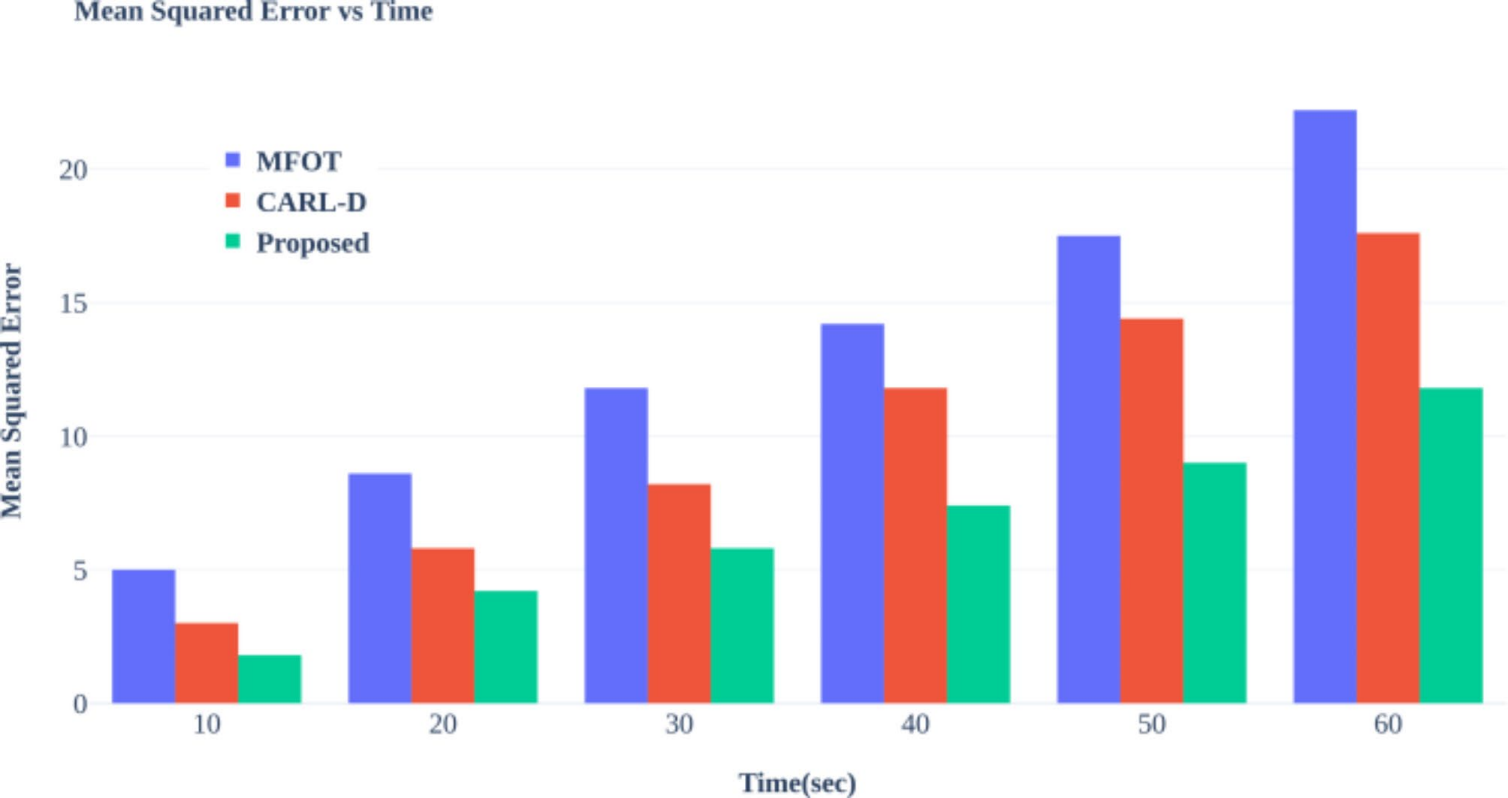
Performance Comparison of Object Tracking Methods: Mean Squared Error vs. Time.

Figure 13. displays the results of the graphical comparative study of mean squared error (MSE) for various approaches over time. Compared to other techniques like MFOTA and CARL-D, the DQN methodology continuously shows a constant decrease in MSE, demonstrating its efficacy in minimizing mistakes. The DQN approach outperforms CARL-D, which achieves 17, and MFOTA, which only drops to 23, to lower the MSE to 12 after 60 s. While the averages for CARL-D and MFOTA are 13.5 and 10.33, respectively, the DQN approach often yields a much lower MSE of 6.51. These results show that the DQN technique is more accurate and dependable than the other examined methods since it constantly maintains a lower MSE.

#### Loss rate

The loss rate tracks the change in the model’s error over multiple training episodes. The study describes the loss rate as a function that ideally declines across episodes

56 $$\:\mathcal{L}\mathcal{R}=f\left(Episode\right)$$

A decreasing loss rate signals improved model performance during training. The DQN method shows a rapid decline in loss rate compared to other methods, further emphasizing its efficient learning and superior performance.

**Fig. 14:**
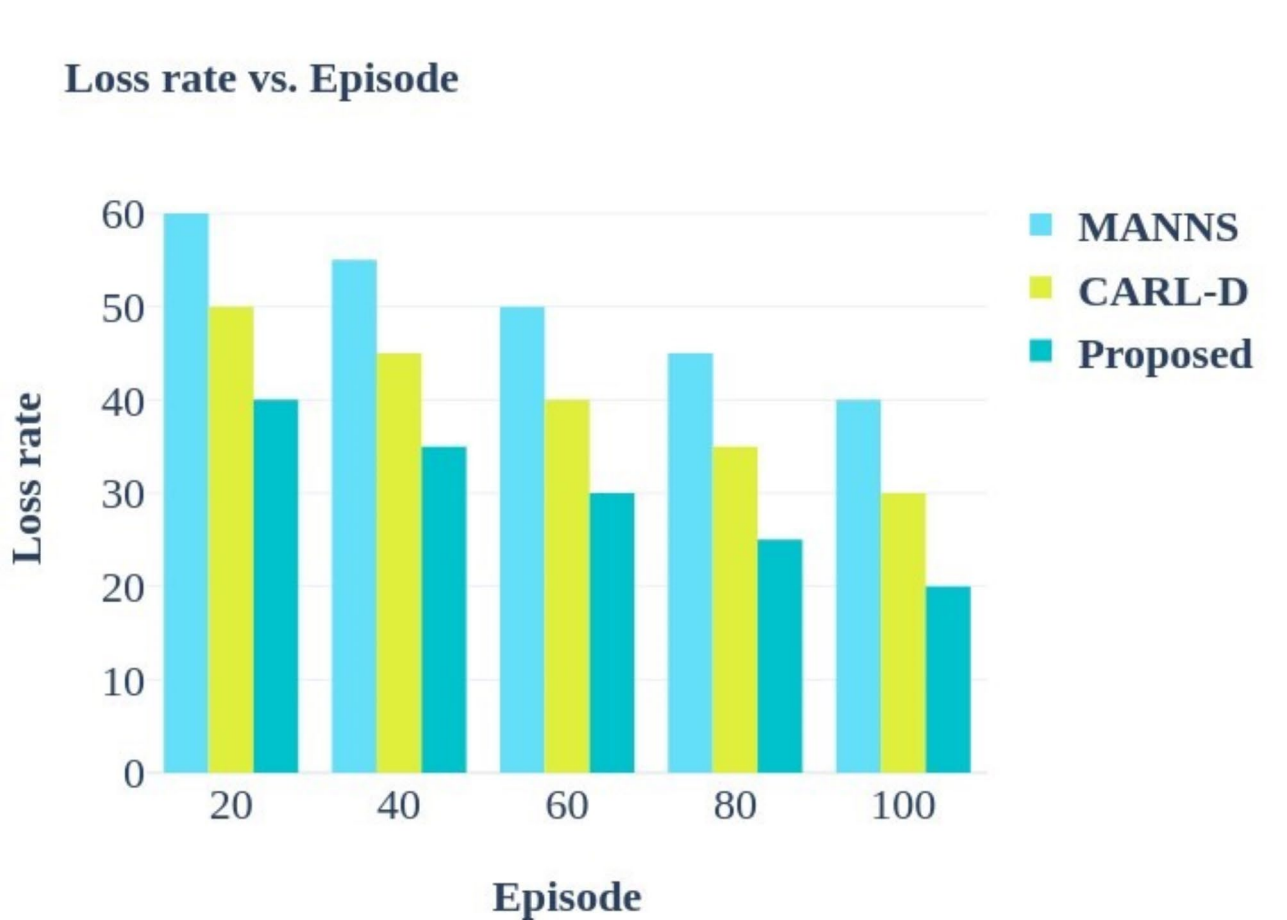
Performance Comparison of Object Tracking Methods: Loss Rate vs. Training Episode.

Figure 14. displays the results of the loss rate graphical comparison study, showing how the DQN strategy successfully lowers the loss rate over time. The suggested strategy exhibits a more notable decrease in loss rate than alternative approaches such as MANNS and CARL-D. In particular, the DQN approach lowers the loss rate to 20 after 100 epochs, while CARL-D lowers it to 30, and MANNS only achieves a loss rate of 40. CARL-D and MANNS average 50 and 40, respectively, whereas the DQN approach maintains an average loss rate of 30. It shows that compared to other methods, the DQN technique minimizes mistakes more effectively since it produces a lower average loss rate.

#### Accumulated reward

Accumulated reward summarizes the total reward the DQN agent has collected across multiple episodes, providing an overall assessment of the learning process. The study expresses it as a function of the episode:

57 $$\:\mathcal{A}\mathcal{R}=f\left(Episode\right)$$

Higher accumulated reward reflects better learning progress. The study shows that the DQN method consistently achieves a lower accumulated reward, illustrating its capacity to efficiently and effectively learn the optimal navigation strategies.

**Fig. 15:**
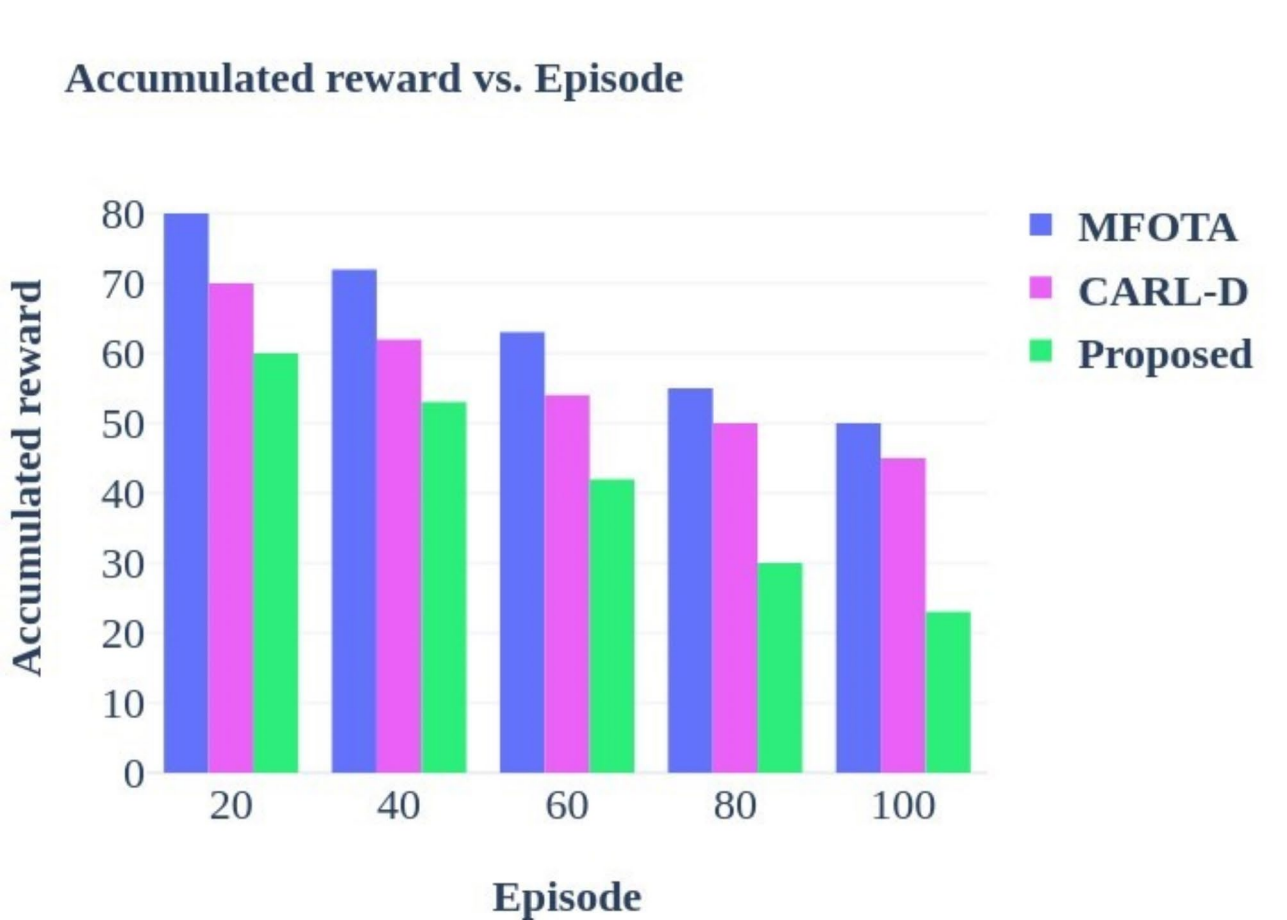
Performance Comparison of Object Tracking Methods: Accumulated Reward vs. Training Episode.

Figure 15. graphically compares the cumulative reward from various methods, demonstrating the effectiveness of the suggested DQN methodology in gradually reducing accumulated rewards over time. The DQN approach reduces the cumulative reward to 23 after 100 epochs, whereas CARL-D and MFOTA only do so to 45 and 50, respectively. The DQN approach has an average cumulative reward of 41.6, but the averages for CARL-D and MFOTA are much higher at 56.2 and 64, respectively. It suggests that, compared to other approaches, the DQN technique is more successful at increasing reward efficiency as it consistently produces a lower average cumulative reward.

### Discussion and limitation

Our proposed DQN-based multi-sensor fusion and segmentation framework demonstrated exceptional performance in challenging autonomous driving scenarios, surpassing existing techniques like CARL-D and MANN. Notably, our model outperformed CARL-D by an average of 14% and achieved a remarkable accuracy rate of 92% while tracking multiple cars. This improvement can be attributed to the successful integration of DenseNet-based fusion and orientation-based segmentation, which enabled accurate object detection even in adverse weather conditions. Furthermore, velocity measurements showed that the DQN model analyzed multi-object tracking data at 18 (H) at 20 km distances, nearly twice as fast as CARL-D. The DQN model’s consistency in high-pressure situations was evident, maintaining an 89% success rate even at speeds of 100 km/h. In contrast, other approaches exhibited lower reliability. These results underscore the potential of our proposed model to enhance the safety and adaptability of autonomous vehicles, particularly in challenging situations where traditional segmentation and tracking techniques fall short.

It is crucial to consider the possible effects of sensor quality and computing limits while talking about the shortcomings of the suggested strategy. The model’s reliance on high-quality LiDAR data is one of its main drawbacks. Environmental elements that cause noise, such as in heavy rain, fog, or snow, can make LiDAR readings less clear and make fusing data from other sensors more difficult. This may decrease object tracking and detection accuracy, particularly during inclement weather. Another constraint is the computing demands of the real-time processing of dense multi-sensor data. It takes a lot of resources to handle high-resolution pictures from several sensors simultaneously, which might cause latency problems and impair the system’s responsiveness in dynamic driving situations. There are also real-world difficulties in implementing this approach on edge devices in autonomous cars. The model’s real-time performance may be compromised by the limited processing power and memory of edge devices, particularly when handling complex, high-speed image data for critical decision-making. This highlights the need for further research in optimizing the model for low-power devices and developing strategies to mitigate the effects of noisy sensor data.

### Research summary

The present research uses deep Q-network (DQN), multi-sensor fusion, and segmentation for multi-object tracking in self-driving cars in adverse weather. We assessed the suggested method in various scenarios to fully validate its resilience and flexibility for autonomous driving. The KITTI dataset simulates the intricacies found in urban and suburban driving settings by offering a wide range of real-world scenarios, such as different weather conditions, traffic volumes, and lighting conditions. To ensure the method’s efficacy throughout weather fluctuations, testing covered scenarios with limited visibility owing to fog and severe rain and sunny and clear circumstances. Furthermore, the assessment covered both low and high traffic densities, enabling the system to show that it can precisely identify objects, segments, and path planning in both open-road and crowded traffic situations. This variety of circumstances gave the method’s multi-sensor fusion and path optimization capabilities a demanding testing environment, confirming its ability to maintain dependable and flexible performance. By tackling these practical issues, the test configuration validates the method’s adaptability and efficiency in attaining secure, effective navigation in intricate settings, which is essential for improving the dependability of autonomous driving. Initially, the KITTI dataset was utilized, and the test images underwent noise removal using the Improved Adaptive Extended Kalman Filter (IAEKF). Subsequent processing involved the improved adaptive weighted mean filter, which adjusted threshold values and corrected missing gaps and tiny regions. Contrast enhancement was achieved through normalized gamma transformation-based CLAHE. Using the LIGHT G Net-based panoptic segmentation methods, the next step involved identifying static infrastructure, such as roads, sidewalks, curbs, lane markers, buildings, and traffic participants. This was followed by image fusion via Dense Net. The integration of offline and online maps was performed using the Energy Valley Optimizer (EVO) to create an online grid map, with path selection conducted using YOLO V7. Multiple objects were tracked based on attributes like position, velocity, color, texture, shape, and location using DQN. The performance of the proposed approach is discussed, with the results of the comparative analysis presented graphically in Figs. 8, 9, 10, 11, 12, 13, 14 and 15 and numerically in Table 1.

**Table 1:** Numerical outcomes.

Performance metrics	MFOTA	MANN	CARL-D	Proposed
Accuracy (%)	-	70	78	92
Velocity(H)	7.8	-	9.5	18
Accuracy rate (%)	-	65	78	97
Success rate (%)	-	57.6	68.4	86.8
Success ratio	0.57	-	0.68	0.88
Mean squared error	23	-	17	12
Loss rate	-	50	40	30
Accumulated reward	50	-	45	23

## Conclusion

This study demonstrated that self-driving cars can achieve effective multi-object tracking in adverse weather conditions through multi-sensor fusion and advanced segmentation techniques. Integrating camera, LiDAR, and radar data enhances perception reliability, enabling safe navigation even in challenging weather. Our proposed approach utilizes multi-sensor fusion and Deep Q Network (DQN)-based segmentation to improve image quality in harsh conditions. The image quality enhancement method, which includes noise removal, contrast enhancement, and adaptive thresholding, significantly boosts segmentation accuracy. By performing multi-segmentation at various optimal angles, we accurately segment narrow and smaller objects within the field of view (FoV) images. Moreover, DenseNet facilitates the fusion of camera, LiDAR, and weather images with FoV data, enhancing the overall quality of service. We constructed a grid map for precise lane and path selection and classified objects as moving or unmoving using YOLO V7, thereby reducing the complexity of reaching the destination. Our approach’s effectiveness was validated through a comparative analysis, yielding a 92% accuracy rate, a velocity of 18 (H), a 97% accuracy in specific scenarios, an 89% success rate, a success ratio of 0.88, a mean squared error of 12 within 60 s, a loss rate of 30, and an accumulated reward of 23. These results indicate that our solution surpasses existing methods across all evaluated metrics. Future research directions include enhancing model resilience to inclement weather, optimizing real-time performance on edge devices, and developing adaptive strategies to maintain accuracy with variable sensor data quality.

## Data Availability

The datasets generated and analyzed during the current study are available online and are publicly accessible at: https://www.cvlibs.net/datasets/kitti/eval_object.php? obj_benchmark=2d.
